# GraphBind: protein structural context embedded rules learned by hierarchical graph neural networks for recognizing nucleic-acid-binding residues

**DOI:** 10.1093/nar/gkab044

**Published:** 2021-02-12

**Authors:** Ying Xia, Chun-Qiu Xia, Xiaoyong Pan, Hong-Bin Shen

**Affiliations:** Institute of Image Processing and Pattern Recognition, Shanghai Jiao Tong University, and Key Laboratory of System Control and Information Processing, Ministry of Education of China, Shanghai 200240, China; Institute of Image Processing and Pattern Recognition, Shanghai Jiao Tong University, and Key Laboratory of System Control and Information Processing, Ministry of Education of China, Shanghai 200240, China; Institute of Image Processing and Pattern Recognition, Shanghai Jiao Tong University, and Key Laboratory of System Control and Information Processing, Ministry of Education of China, Shanghai 200240, China; Institute of Image Processing and Pattern Recognition, Shanghai Jiao Tong University, and Key Laboratory of System Control and Information Processing, Ministry of Education of China, Shanghai 200240, China; School of Life Sciences and Biotechnology, Shanghai Jiao Tong University, Shanghai 200240, China

## Abstract

Knowledge of the interactions between proteins and nucleic acids is the basis of understanding various biological activities and designing new drugs. How to accurately identify the nucleic-acid-binding residues remains a challenging task. In this paper, we propose an accurate predictor, GraphBind, for identifying nucleic-acid-binding residues on proteins based on an end-to-end graph neural network. Considering that binding sites often behave in highly conservative patterns on local tertiary structures, we first construct graphs based on the structural contexts of target residues and their spatial neighborhood. Then, hierarchical graph neural networks (HGNNs) are used to embed the latent local patterns of structural and bio-physicochemical characteristics for binding residue recognition. We comprehensively evaluate GraphBind on DNA/RNA benchmark datasets. The results demonstrate the superior performance of GraphBind than state-of-the-art methods. Moreover, GraphBind is extended to other ligand-binding residue prediction to verify its generalization capability. Web server of GraphBind is freely available at http://www.csbio.sjtu.edu.cn/bioinf/GraphBind/.

## INTRODUCTION

Interactions between proteins and nucleic acids participate in various biological activities and processes, such as gene replication and expression, signal transduction, regulation and metabolism (1–3). Studying the interactions between proteins and nucleic acids is important for analyzing genetic material, understanding protein functions and designing new drugs. Many experimental methods, such as X-ray, nuclear magnetic resonance spectroscopy and laser Raman spectroscopy, are designed to solve the native structures of complexes to investigate molecular interactions. However, they are usually time-consuming and costly. It is highly desirable to develop reliable and accurate computational methods for recognizing nucleic-acid-binding residues in a large-scale screening manner (4).

Existing computational methods for recognizing nucleic-acid-binding residues can be generally divided into two groups according to the used data types: sequence-based and structure-based methods. Sequence-based methods, such as ConSurf (5), TargetDNA (6), DRNApred (4), SCRIBER (3) and TargetS (7), learn local patterns of bio-physicochemical characteristics using sequence-derived features. For example, in TargetDNA, evolutionary conservative information and predicted solvent accessibility of proteins are extracted from protein sequences and SVMs are used to identify DNA-binding residues from their sequence contexts which are determined by a sliding window strategy (6). The advantage of sequence-based methods is that they can perform a prediction for any protein from its sequence alone. However, their performance could be limited since the potential patterns of binding residues are not evident from their sequences alone, but are conserved in spatial structures (8,9). Thus, the features captured from protein sequences might not be sufficient to represent residues accurately.

Different from sequence-based methods, the assumption of the structure-based methods is that structural motifs with specific functions often behave in highly conservative patterns on local tertiary structures (8,9). The structure-based methods can be categorized into the following two types: (i) template-based methods, such as DR_bind1 (10) and TM-SITE (11), which search for reliable templates for query proteins by structure comparison and infer interactions between the proteins and nucleic acids according to the principles of physics and chemistry; (ii) feature-based machine learning methods, such as aaRNA (12) and NucleicNet (13), which construct classifiers with features derived from protein structures.

Functional sites are frequently determined by the local patterns of tertiary structures beyond sequences (14). We focus on identifying nucleic-acid-binding residues from protein structures with feature-based machine learning methods. One major challenge is how to embed the crucial structural and bio-physicochemical characteristics for downstream binding residue recognition. Previous methods usually use hand-crafted features to represent structures (12). These methods require strong domain knowledge, and the hand-crafted features may fail to capture critical information of proteins for specific downstream tasks. Some other methods encode protein structures into a three-dimensional (3D) Euclidean space(15,16). For example, DeepSite maps protein atoms into 3D voxels to represent the protein structures (16). Then 3D convolutional neural networks (3DCNNs) (17) are used to extract abstract features of target residues from their neighborhood based on the 3D volumetric representation (16). There are two potential disadvantages in 3D volumetric representation of protein structures: (i) the sparse and irregular distribution of residues makes it difficult to represent the neighborhood information of residues and (ii) it is difficult to guarantee the invariance of rotation and translation in the 3D Cartesian coordinate system. Alternately, DELIA calculates a distance matrix to represent the distance relationship of the residue pairs. DELIA treats the structures as 2D images and uses fixed-size convolution kernels (18) to learn patterns from local distance relationship for all residues (19), resulting in incomplete neighborhood information for some residues and ignoring the knowledge passing between structural adjacent residues.

To better capture the protein structure information and the spatial relationships among residues, graphs are employed to represent the protein structures, where nodes represent residues and edges are defined according to the spatial relationships among residues. The graph representation can not only be invariant to rotation and translation, but also handle the varying number of the unordered neighbors of residues. Recently, graph neural networks (GNNs) have emerged as powerful tools for graph data in computational biology (20). For example, Fout *et al.* present a GNN-based method for classifying pairwise residue interactions from protein structures (21). Decagon predicts the side effects of different drug combinations using graph convolutional networks (GCNs) (22). DimiG infers microRNA-associated diseases on an interaction graph using semi-supervised GCNs (23). Torng and Altman propose a two-step graph-convolutional (Graph-CNN) framework for predicting drug-target interactions (24). All the above studies demonstrate that GNNs are effective in processing the biological and chemical graph data.

In this study, we propose an accurate nucleic-acid-binding residues predictor, GraphBind, based on the graphs constructed from structural contexts and hierarchical graph neural networks (HGNNs). To extract the crucially local patterns of structural and bio-physicochemical characteristics from protein structures, for each target residue, we first construct a graph based on the local environment of the target residue. Initial node feature vectors consist of evolutionary conservation, secondary structure information, other bio-physicochemical characteristics and position embeddings. Position embeddings are calculated from geometric knowledge that defines spatial relationship of residues in the structural context. Initial edge feature vectors are also derived from the geometric knowledge. Then, we construct a hierarchical graph neural network to learn the latent local patterns for binding residue prediction. Edge update module, node update module and graph update module are designed to learn the high-level geometric and bio-physicochemical characteristics as well as a fixed-size embedding of the target residue. In addition, gated recurrent units (25) are used to stack multiple GNN-blocks, which take advantage of all blocks’ information and avoid the gradient vanishing problem. The experimental results demonstrate the superior performance of GraphBind on nucleic-acid-binding residue prediction. Moreover, we demonstrate that GraphBind can be extended to other ligand-binding residue prediction with promising performance.

## MATERIALS AND METHODS

In this section, two benchmark datasets are constructed to evaluate the performance of GraphBind. Then, graph construction and architecture of HGNNs are introduced. Finally, evaluation protocol and detailed experimental settings are summarized briefly.

### Benchmark datasets

To evaluate the performance of GraphBind and fairly compare it with other methods, we construct two nucleic-acid-binding protein benchmark datasets from the BioLiP database (26) and split them into training and test sets according to the release date. The benchmark datasets are available at http://www.csbio.sjtu.edu.cn/bioinf/GraphBind/datasets.html.

The DNA/RNA-binding proteins are collected from the BioLiP database, released on 5 December 2018. This database is a collection of biologically relevant ligand-protein interactions that are solved structurally in complexes. If the smallest atomic distance between the target residue and the nucleic acid molecule is less than 0.5 Å plus the sum of the Van der Waal's radius of the two nearest atoms, it will be defined as a binding residue.

BioLiP contains 48133 nucleic-acid-binding sites from 6342 nucleic-acid-protein complexes in 5 December 2018. These complexes are divided into 4344 DNA-protein complexes (9574 DNA-binding protein chains), 1558 RNA-protein complexes (7693 RNA-binding protein chains) and 440 DNA-RNA-protein complexes. We exclude the DNA-RNA-protein complexes to avoid confusion since no annotation is made to distinguish DNA- or RNA-binding residues in the BioLip database. According to the release date, protein chains released before 6 January 2016, are assigned into original training sets (6731 DNA-binding protein chains and 6426 RNA-binding protein chains), while the remaining protein chains are assigned into original test sets (2843 DNA-binding protein chains and 1267 RNA-binding protein chains).

DNA/RNA-binding residue prediction suffers from the data imbalance problem that the number of DNA/RNA-binding residues is much smaller than the number of non-binding residues, so we apply data augmentation on the original training sets. Following previous studies (3,4,27–29), we transfer binding annotations from similar protein chains to increase the number of binding residues in the training sets for the following reasons: (i) proteins with similar sequences and structures, although could derived from different organisms, may have the same biological function; (ii) different resolutions may lead to minor differences in the structure for the same protein. To this end, we first apply bl2seq (30) (*E*-value < 0.001) and TM-align (31) to assess the sequence identity and structural similarity between protein chain pairs. Second, we cluster the chains that have sequence identity >0.8 and TM scores >0.5. Third, the annotations of protein chains in the same cluster are transferred into the chain that has the largest number of residues. After transferring binding annotations, we further remove the redundant protein chains with CD-HIT (32) to reduce the sequence identity in the training set to be less than 30%. Finally, we obtain 573 DNA-binding and 495 RNA-binding protein chains which are served as the training sets. The data augmentation increases the numbers of DNA- and RNA-binding residues by 30.7% and 24.3%, respectively. Protein chains from the original DNA/RNA-binding test set with over 30% sequence identity measured by CD-HIT (32) to any chain in the DNA/RNA-binding training set are removed. Finally, we obtain 129 DNA-binding proteins and 117 RNA-binding proteins as the DNA- and RNA-binding test sets, respectively. The details of the datasets are summarized in Table 1 (see Supplementary Table S1 for training sets without data augmentation).

**Table 1.** Summary of the benchmark datasets

Type	Dataset	*N*_protein_^a^	*N*_pos_^b^	*N*_neg_^c^	PNratio^d^
DNA	DNA-573_Train	573	14479	145404	0.100
	DNA-129_Test	129	2240	35275	0.064
RNA	RNA-495_Train	495	14609	122290	0.119
	RNA-117_Test	117	2031	35314	0.058

^a^Number of proteins.

^b^Number of binding residues.

^c^Number of non-binding residues.

^d^PNratio = *N*_pos_/*N*_neg_.

### Graph construction based on structural contexts

**Figure 1.**
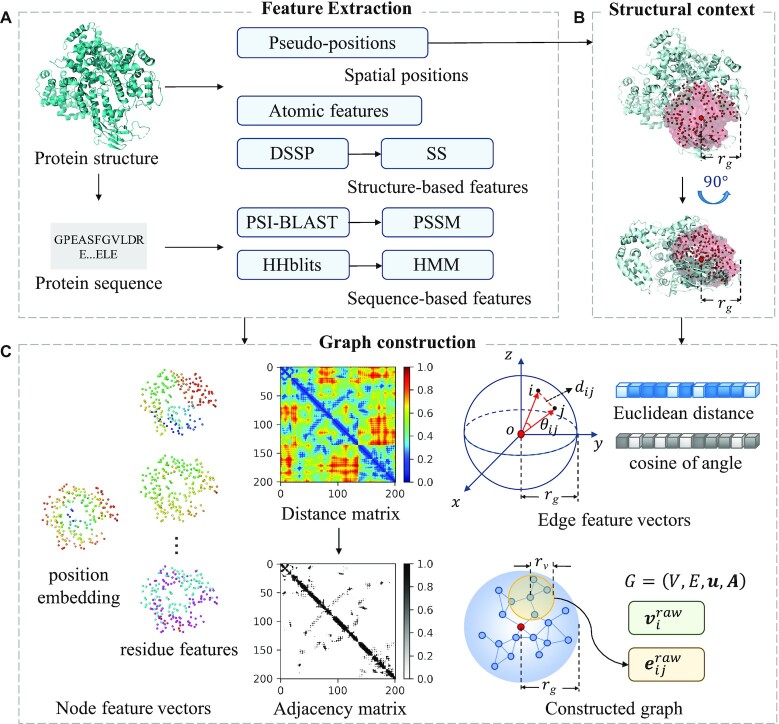
Pipeline of graph construction used in GraphBind. It consists of three modules: feature extraction, structural context extraction and graph construction. (**A**) Feature extraction. Pseudo-positions and atomic features of residues are extracted from protein structures. DSSP, PSI-BLAST and HHblits are employed to extract secondary structure profiles and evolutionary conversation profiles from protein structures and sequences. (**B**) Structural context extraction. The structural context of a target residue is determined by a sliding sphere of a predefined radius ${r_g}$ centering at the residue. (**C**) Graph construction. The structural context is further represented by a graph $G\ = ( {V,\ E,\ \boldsymbol{u},\ \boldsymbol{A}} )$. *V*, *E*, ***u*** and ***A*** denote the set of feature vectors of nodes, the set of feature vectors of edges, the graph feature vector and the adjacency matrix, respectively. Nodes in the graph represent residues. The raw feature vector $\boldsymbol{v}_i^{raw} \in {\mathbb{R}^{72}}$ of node $i$ is the concatenation of the position embedding and the residue features of node *i*. Distance matrix is calculated based on pseudo-positions of residues. We apply the binary threshold ${r_v}$ on the distance matrix to get the adjacency matrix ***A***, which records the connections of nodes. The raw feature vector $\boldsymbol{e}_{ij}^{raw} \in {\mathbb{R}^2}$ of edge $ij$ is encoded by the Euclidean distance between the two adjacent nodes, and the cosine of the angle ${{\rm{\theta }}_{ij}}$ between the two vectors from the sphere center to the two adjacent nodes, respectively.

Multiple types of sequence-based and structure-based features are extracted, including pseudo-positions, atomic features of residues, secondary structure profiles and evolutionary conversation profiles. Then, a sliding sphere defined in the 3D space is used to extract the structural context for the target residue centering at the residue. The adjacent matrix calculated based on the pseudo-positions of residues in the structural context is used to construct the graph. Besides, the geometric knowledge and bio-physicochemical characteristics are embedded in node and edge feature vectors. The pipeline of graph construction is shown in Figure 1.

#### Feature extraction

Four types of residue-level features are derived as following:

The first is pseudo-positions. The centroid of a residue including both backbone and side-chain atoms of the residue is denoted as the pseudo-position of this residue since interactions between proteins and nucleic acids can occur on both backbone and side-chain atoms (33).

The second is atomic features of residues. For a residue, we extract the following seven kinds of features of each atom belonging to the residue (excluding hydrogen atoms): atom mass, *B*-factor, whether it is a residue side-chain atom, electronic charge, the number of hydrogen atoms bonded to it, whether it is in a ring, and the van der Waals radius of the atom. The original atomic features of a residue are denoted as ${\{ {{f_{s,t}}} \}_{s\ = \ 1, \ldots ,7,\ t\ = \ 1, \ldots ,{N_a}}}$, where${f_{s,t}}$ stands for the *s*th feature of *t*th atom and ${N_a}$ stands for the number of atoms belonging to the residue. Since different residues may have different numbers of atoms, we average the *s*th feature of all the atoms as the processed *s*th atomic feature ${x_s}$ of the residue, which results in seven kinds of features for each residue ${\{ {{x_s}} \}_{s\ = \ 1, \ldots ,7}}$:

(1) $$\begin{equation*}{x_s} = \frac{1}{{{N_a}}}\ \left( {\mathop \sum \limits_{t\ = {\rm{\ }}1}^{t\ =\ {N_a}} {f_{s,t}}} \right)\end{equation*}$$

Finally, we generate an atomic feature matrix with the size of $L \times$7 for the query protein with $L$ residues.

The third is secondary structure profile. DSSP (34,35) generates the secondary structure profile as a matrix with the size of $L \times$14, including one column of residue water-exposed surface, five columns of bond and torsion angles and eight columns of one-hot encoded secondary structure with eight states. The eight states of secondary structure contain B(residue in isolated ${\rm{\beta }}$-bridge), E(extended strand, participates in ${\rm{\beta }}$-ladder), G(3_10_-helix), H(${\rm{\alpha }}$-helix), I(${\rm{\pi }}$-helix), S(bend), T(H-bonded turn) and others.

The last is two evolutionary conversation profiles.

1. PSI-BLAST profile. The alignment tool PSI-BLAST applies the heuristic algorithms and dynamic programming to search the NCBI’s non-redundant database (NR) for homologous sequences with three iterations and *E*-value < 10^−3^ (36). The size of the generated position-specific scoring matrix (PSSM) is ${\rm{\ }}L \times$20. Each element *x* in the PSSM is normalized to the range [0, 1] by a sigmoid function:
  (2) $$\begin{equation*}\bar{x} = \frac{1}{{1 + {e^{ - x}}}}\ \end{equation*}$$
2. HHblits profile. HHblits, which is based on hidden Markov models (HMMs), is used to search against the uniclust30 database with default parameters to generate HMM matrix for the query sequence(37). The size of the HMM matrix is $L \times$30. The HMM matrix consists of 20 columns of observed frequencies for 20 amino acids in homologous sequences, seven columns of transition frequencies and three columns of local diversities. Each score is converted to the range [0, 1]:
  (3) $$\begin{equation*}\bar{x} = \frac{x}{{10000}}\ \end{equation*}$$

The PSI-BLAST and HHblits profiles are complementary since their backend algorithms and searched databases are different, which is confirmed in our following experiments.

In summary, for a query protein, we obtain the pseudo-position matrix with the size of $L \times$3 and a feature matrix with the size of $L \times$71. For each column in the feature matrix, the min-max normalization is carried out to linearly normalize the value to [0, 1]:

(4) $$\begin{equation*}\bar{x} = \frac{{x - {x_{{\rm min}}}}}{{{x_{{\rm max}}} - {x_{{\rm min}}}}}\ \end{equation*}$$

where ${x_{{\rm min}}}$ and ${x_{{\rm max}}}$ are the minimum and the maximum values of this feature in the training set, respectively.

#### Structural context extraction

According to the pseudo-positions of residues in the tertiary structure, a sphere slides along the polypeptide chain to obtain the structural context for each residue. For a target residue, the structural context is defined as a sphere with a radius ${r_g}$ centering at this residue. All residues in the sphere and their geometric knowledge form the local structural context of the target residue. Compared to the overall structure of a protein, the binding site is usually more related to the geometric and bio-physicochemical properties of its local structural environment (8–9,15).

#### Graph construction

In this step, the structural context of a residue is further represented as a graph. A graph is defined as $G\ = ( {V,\ E,\ {\boldsymbol{u}},\ {\boldsymbol{A}}} )$, where $V\ = {\{ {{{\boldsymbol{v}}_i}} \}_{i = 1, \ldots ,{N_v}}}$ and ${{\boldsymbol{v}}_i} \in {\mathbb{R}^{{D_v}}}$ denote the set of feature vectors of ${N_v}$ nodes and the feature vector of node $i$, respectively. ${\boldsymbol{A}}$ denotes the adjacency matrix with the shape of ${N_v} \times {N_v}$. $E\ = \{ {{{\boldsymbol{e}}_{ij}}|{{\boldsymbol{A}}_{ij}} = 1} \}$ denotes the set of feature vectors of ${N_e}$ edges. ${{\boldsymbol{e}}_{ij}} \in {\mathbb{R}^{{D_e}}}$ stands for the feature vector of the edge $ij$ between node $i$ and $j$. ${{\boldsymbol{e}}_{ij}} \in E$ if ${{\boldsymbol{A}}_{ij}} = \ 1$, ${{\boldsymbol{e}}_{ij}} \notin E$ if ${{\boldsymbol{A}}_{ij}} = \ 0$. ${\boldsymbol{u}}$ stands for the graph feature vector. In the graph, a residue is denoted as a node. Position of *i*th node ${{\boldsymbol{p}}_i}$ is defined by the pseudo-position of the corresponding residue. Residues around target residues may form specific local geometric patterns which are informative for binding residue recognition. Motivated by this observation, we use position embedding to represent the positional relationship between the target residue and each of its contextual residues since it contains local geometric knowledge around the target residue. The position embedding of node $i$ is defined as the normalized Euclidean distance between node$\ i$$\ i$ and the sphere center,

(5) $$\begin{equation*}{PE_i} = \frac{1}{{{r_g}}}\ \left| {\overrightarrow {{{\boldsymbol{p}}_o}{{\boldsymbol{p}}_i}} } \right|\end{equation*}$$

where ${{\boldsymbol{p}}_o}$ and ${{\boldsymbol{p}}_i}$ respectively stand for the position of the sphere center and node $i$, and $\overrightarrow {{{\boldsymbol{p}}_o}{{\boldsymbol{p}}_i}}$ is the vector from ${{\boldsymbol{p}}_o}$ to ${{\boldsymbol{p}}_i}$. The raw feature vector ${\boldsymbol{v}}_i^{raw} \in {\mathbb{R}^{72}}$ of node $i$ is the concatenation of the position embedding ${PE_i}$ and the 71 residue features of the node. The set of raw node feature vectors is denoted as ${V^{raw}} = {\{ {{\boldsymbol{v}}_i^{raw}} \}_{i = 1, \ldots ,{N_v}}}$.

Then, a distance matrix ${\boldsymbol{D}}$ with the size of ${N_v} \times {N_v}$ is constructed. The element ${{\boldsymbol{D}}_{ij}}$ is the Euclidean distance between node $i$ and node$\ j$:

(6) $$\begin{equation*}{{\boldsymbol{D}}_{ij}} = \left| {\overrightarrow {{{\boldsymbol{p}}_i}{{\boldsymbol{p}}_j}} } \right|\ \end{equation*}$$

We use a threshold ${r_v}$ on ${\boldsymbol{D}}$ to get the adjacency matrix ${\boldsymbol{A}}$,

(7) $$\begin{equation*}{{\boldsymbol{A}}_{ij}} = \{ \begin{array}{@{}*{1}{c}@{}} {1,{\rm{\ }}if{\rm{\ }}{{\boldsymbol{D}}_{ij}} < {r_v}}\\ {0,if{\rm{\ }}{{\boldsymbol{D}}_{ij}} \ge {r_v}} \end{array}\ \end{equation*}$$

The value of ${r_v}$ is selected based on the validation set.

The raw feature vector of edge $ij$ is denoted as ${\boldsymbol{e}}_{ij}^{raw} \in {\mathbb{R}^2}$, which consists of two properties related to the geometric knowledge: (i) the Euclidean distance ${{\boldsymbol{D}}_{ij}}$ of node $i$ and node$\ j$$\ j$, and (ii) the cosine of the angle ${{\rm{\theta }}_{ij}}$ between the two vectors $\overrightarrow {{{\boldsymbol{p}}_o}{{\boldsymbol{p}}_i}}$ and $\overrightarrow {{{\boldsymbol{p}}_o}{{\boldsymbol{p}}_j}}$, which are vectors respectively from the sphere center to the node $i$ and node$\ j$$\ j$:

(8) $$\begin{equation*}{\rm cos}\left( {{{\rm{\theta }}_{ij}}} \right) = \frac{{\overrightarrow {{{\boldsymbol{p}}_o}{{\boldsymbol{p}}_i}} \cdot \overrightarrow {{{\boldsymbol{p}}_o}{{\boldsymbol{p}}_j}} }}{{\left| {\overrightarrow {{{\boldsymbol{p}}_o}{{\boldsymbol{p}}_i}} } \right|\left| {\overrightarrow {{{\boldsymbol{p}}_o}{{\boldsymbol{p}}_j}} } \right|}}\ \end{equation*}$$

where $ \cdot$ means dot product. ${\boldsymbol{e}}_{ij}^{raw}$ is also normalized to [0, 1]. The set of raw edge feature vectors is denoted as ${E^{raw}} = \{ {{\boldsymbol{e}}_{ij}^{raw}|{{\boldsymbol{A}}_{ij}} = 1} \}$. It is worth noting that all position-related features of nodes and edges are defined in terms of the relative distance between nodes. Thus, GraphBind is invariant to rotation and translation.

### Hierarchical graph neural networks

**Figure 2.**
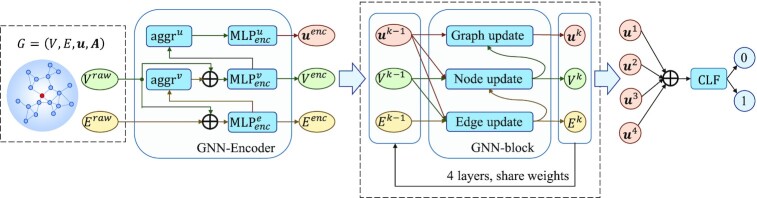
Diagram of the hierarchical graph neural network (HGNN). The HGNN consists of a GNN-Encoder, four GRU-based GNN-blocks and a multilayer perceptron classifier (CLF). The GNN-Encoder encodes the set of raw node feature vectors ${V^{raw}}$ and the set of raw edge feature vectors ${E^{raw}}$ of the graph into the high-level representations ${V^{enc}}$ and ${E^{enc}}$, and calculates the graph feature vector ${{\boldsymbol{u}}^{enc}}$ from the set of the encoded node feature vectors ${V^{enc}}$. The stacked four GNN-blocks hierarchically update the latent feature vectors of edges, nodes and graph. Four fixed-size graph feature vectors ${\{ {{{\boldsymbol{u}}^k}} \}_{k = 1, \ldots ,4}}$ are obtained. Finally, ${\{ {{{\boldsymbol{u}}^k}} \}_{k = 1, \ldots ,4}}$ are concatenated to be fed into the CLF for binding residue prediction.

After constructing the graph of each residue with geometric knowledge and bio-physicochemical characteristics, a hierarchical graph neural network (HGNN) is designed to embed the graph to a fixed-size graph-level latent representation for downstream prediction. The HGNN consists of three modules. (i) A graph neural network encoder (GNN-Encoder). It is designed for encoding the set of raw edge and node feature vectors into the high-level representations and calculating the graph feature vector from the set of the encoded node feature vectors. (ii) The gated-recurrent-unit-based graph neural network blocks (GNN-blocks). Four GNN-blocks are stacked to expand the range of receptive fields and hierarchically update the latent feature vectors of edges, nodes and graph. Each GNN-block embeds the structural context into a fixed-size graph feature vector. (iii) A multilayer perceptron classifier (CLF). It is applied for classifying binding residues with the concatenated vector from the above four graph feature vectors. The diagram of the HGNN is shown in Figure 2.

Here, we first introduce two basic operations, multilayer perception (MLP) and gated recurrent unit (GRU).

1. MLP. MLP is a point-by-point nonlinear transformation defined in the Eq. (9), which consists of two linear layers and a rectified linear unit (ReLU) (38):
  (9) $$\begin{equation*}{\rm{MLP\ }}\left( {\boldsymbol{X}} \right) = {{\boldsymbol{W}}_2}{\rm{\ max}}\left( {0,{{\boldsymbol{W}}_1}{\boldsymbol{X}} + {{\boldsymbol{b}}_1}} \right) + {{\boldsymbol{b}}_2}\end{equation*}$$
2. GRU (25). GRU is widely used in natural language processing for text sequences. It does not erase the previous information over time, but retains the relevant information and passes it to the next unit by nonlinearly weighting the inputs and the hidden states to inference the outputs. GRU takes advantage of all units’ information and avoids gradient vanishing. For each time step $t$, based on the input ${{\boldsymbol{X}}^t}$ and the previous hidden state ${{\boldsymbol{h}}^{t - 1}}$, the output of GRU is calculated by:
  (10) $$\begin{equation*}{r_t} = {\rm{\ }}\sigma \left( {{{\boldsymbol{W}}_r}{{\boldsymbol{X}}^t} + {{\boldsymbol{U}}_r}{{\boldsymbol{h}}^{t - 1}}} \right)\end{equation*}$$
  (11) $$\begin{equation*}{z_t} = {\rm{\ }}\sigma \left( {{{\boldsymbol{W}}_z}{{\boldsymbol{X}}^t} + {{\boldsymbol{U}}_z}{{\boldsymbol{h}}^{t - 1}}} \right)\end{equation*}$$
  (12) $$\begin{equation*}{{\boldsymbol{\tilde{h}}}^t} = {\rm{\ tanh}}\left( {{{\boldsymbol{W}}_{\tilde{h}}}{{\boldsymbol{X}}^t} + {{\boldsymbol{U}}_{\tilde{h}}}\left( {{r_t} \cdot {{\boldsymbol{h}}^{t - 1}}} \right)} \right)\end{equation*}$$
  (13) $$\begin{equation*}{{\boldsymbol{h}}^t} = {z_t}\ {{\boldsymbol{h}}^{t - 1}} + \left( {1 - {z_t}} \right){{\boldsymbol{\tilde{h}}}^t}\end{equation*}$$
  where $\sigma$ is the sigmoid activation function and $ \cdot$ means dot product. ${r_t}$ is the reset gate, which determines that how much information from the previous hidden state ${{\boldsymbol{h}}^{t - 1}}$ can be conveyed. ${z_t}$ is the update gate, which determines the proportion of the previously hidden state ${{\boldsymbol{h}}^{t - 1}}$ and the new hidden state ${{\boldsymbol{\tilde{h}}}^t}$ in the updated hidden state ${{\boldsymbol{h}}^t}$ (25).

#### GNN-Encoder

GNN-Encoder encodes the set of raw node feature vectors ${V^{raw}}$ and the set of raw edge feature vectors ${E^{raw}}$ into the high-level representations of the nodes ${V^{enc}} = {\{ {{\boldsymbol{v}}_i^{enc}} \}_{i = 1, \ldots ,{N_v}}}$, edges ${E^{enc}} = \{ {{\boldsymbol{e}}_{ij}^{enc}|{{\boldsymbol{A}}_{ij}} = 1} \}$ and graph ${{\boldsymbol{u}}^{enc}}$. ${\boldsymbol{v}}_i^{enc} \in {\mathbb{R}^{{D_v}}}$,${\boldsymbol{\ e}}_{ij}^{enc} \in {\mathbb{R}^{{D_e}}}$ and ${{\boldsymbol{u}}^{enc}} \in {\mathbb{R}^{{D_u}}}$.

First, the encoded edge feature vector ${\boldsymbol{e}}_{ij}^{enc}$ is calculated from the raw edge feature vector ${\boldsymbol{e}}_{ij}^{raw}$ and the raw node feature vectors ${\boldsymbol{v}}_i^{raw}$ and ${\boldsymbol{v}}_j^{raw}$:

(14) $$\begin{equation*}{\boldsymbol{e}}_{ij}^{enc} = {\rm{MLP}}_{enc}^e\ \left( {\left[ {{\boldsymbol{e}}_{ij}^{raw};{\boldsymbol{v}}_i^{raw};{\rm{\ }}{\boldsymbol{v}}_j^{raw}} \right]} \right)\end{equation*}$$

where ${\rm{MLP}}_{enc}^e$ is an MLP operation to perform nonlinear transformation, and $\left[ {{\boldsymbol{e}}_{ij}^{raw};{\boldsymbol{v}}_i^{raw};{\rm{\ }}{\boldsymbol{v}}_j^{raw}} \right]$ means the concatenation of ${\boldsymbol{e}}_{ij}^{raw}$, ${\boldsymbol{v}}_{i}^{raw}$ and ${\boldsymbol{v}}_{j}^{raw}$.

Next, the node feature vector ${\boldsymbol{v}}_i^{enc}$ is updated from the raw node feature vector ${\boldsymbol{v}}_i^{raw}$ and the sum aggregation of the above updated feature vectors of its adjacent edges:

(15) $$\begin{equation*}{\boldsymbol{v}}_i^{enc} = {\rm{MLP}}_{enc}^v\ \left( {\left[ {{\rm{\ }}{\boldsymbol{v}}_i^{raw};{\rm{\ }}\mathop \sum \limits_{j \in N\left( {{v_i}} \right)} {\boldsymbol{e}}_{ij}^{enc}} \right]} \right)\end{equation*}$$

where $N( {{v_i}} )$ is the set of neighbors of node $i$, and ${\rm{MLP}}_{enc}^v$ is an MLP operation to perform nonlinear transformation.

Finally, the graph feature vector ${{\boldsymbol{u}}^{enc}}$ is obtained by performing nonlinear transformation on the sum of the set of encoded node feature vectors in this graph:

(16) $$\begin{equation*}{{\boldsymbol{u}}^{enc}} = {\rm{MLP}}_{enc}^u\ \left( {\mathop \sum \limits_{i\ = {\rm{\ }}1}^{{N_v}} {\boldsymbol{v}}_i^{enc}} \right)\end{equation*}$$

where ${\rm{MLP}}_{enc}^u$ is an MLP operation to perform nonlinear transformation.

#### Stacked multiple GNN-blocks

**Figure 3.**
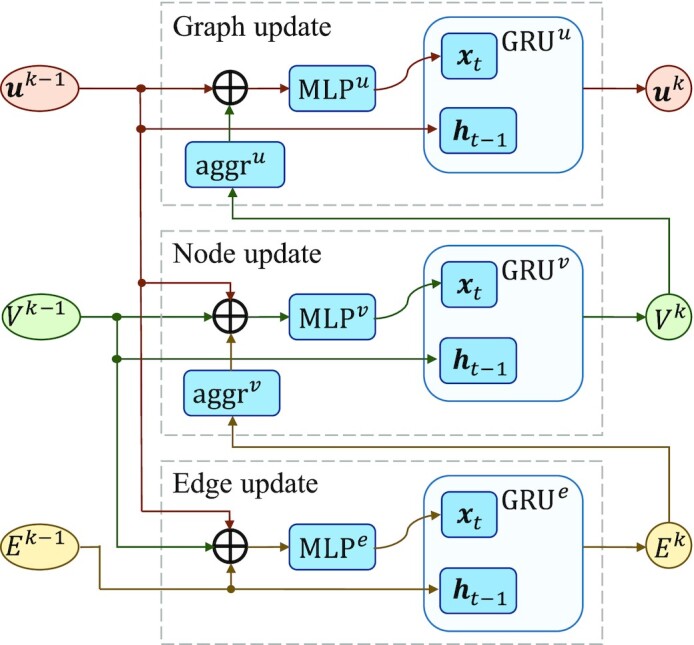
The GNN-block updates edge, node and graph feature vectors sequentially. The GRUs weight the outputs of the layer $k$ and the outputs of the layer $k - 1$ to control the propagation range of edges, nodes and graphs.

Similar to CNNs, the receptive field can be expanded by stacking multiple GNN-blocks. Thus, the remote edges or nodes can affect each other until their latent representations reach stability (39). A GNN-block updates edge, node and graph feature vectors sequentially, as shown in Figure 3.

1. Edge update. We first calculate the intermediate edge feature vector ${\boldsymbol{e}}_{ij}^{k^{\prime}}$ of the layer $k$, which takes the concatenation of the edge feature vector ${\boldsymbol{e}}_{ij}^{k - 1}$, the two node feature vectors ${\boldsymbol{v}}_i^{k - 1}$ and ${\boldsymbol{v}}_j^{k - 1}$, and the graph feature vector ${{\boldsymbol{u}}^{k - 1}}$ of the previous layer as input. The input is fed into the nonlinear transformation ${\rm{ML}}{{\rm{P}}^e}$ to get the intermediate output ${\boldsymbol{e}}_{ij}^{k^{\prime}}$, and ${\rm{GR}}{{\rm{U}}^e}{\rm{\ }}$is used to perform nonlinear weighted transformation. The updated edge feature vector ${\boldsymbol{e}}_{ij}^k$ of the layer $k$ is derived as following:
  (17) $$\begin{equation*}{\boldsymbol{e}}_{ij}^{k^{\prime}} = {\rm{ML}}{{\rm{P}}^e}\ \left( {\left[ {{\boldsymbol{e}}_{ij}^{k - 1};{\boldsymbol{v}}_i^{k - 1};{\rm{\ }}{\boldsymbol{v}}_j^{k - 1};{{\boldsymbol{u}}^{k - 1}}} \right]} \right)\end{equation*}$$
  (18) $$\begin{equation*}{\boldsymbol{e}}_{ij}^k = {\rm{GR}}{{\rm{U}}^e}\ \left( {{\boldsymbol{e}}_{ij}^{k^{\prime}},{\rm{\ }}{\boldsymbol{e}}_{ij}^{k - 1}} \right)\end{equation*}$$
2. Node update. We aggregate the updated feature vectors of the adjacent edges of node $i$ as its neighbor edge feature vector. The intermediate output ${\boldsymbol{v}}_i^{k^{\prime}}$ is nonlinearly transformed by ${\rm{ML}}{{\rm{P}}^v}$ on the concatenation of its neighboring edge feature vectors, node feature vector ${\boldsymbol{v}}_i^{k - 1}$ and the graph feature vector ${{\boldsymbol{u}}^{k - 1}}$. Then, ${\rm{GR}}{{\rm{U}}^v}$ weights ${\boldsymbol{v}}_i^{k^{\prime}}$ and ${\boldsymbol{v}}_i^{k - 1}$ to obtain the updated node feature vector ${\boldsymbol{v}}_i^k$:
  (19) $$\begin{equation*}{\boldsymbol{v}}_i^{k^{\prime}} = {\rm{ML}}{{\rm{P}}^v}\ \left( {\left[ {{\rm{\ }}{\boldsymbol{v}}_i^{k - 1};{\rm{\ }}\mathop \sum \limits_{j \in N\left( {{v_i}} \right)} {\boldsymbol{e}}_{ij}^k;{{\boldsymbol{u}}^{k - 1}}} \right]} \right)\end{equation*}$$
  (20) $$\begin{equation*}{\boldsymbol{v}}_i^k = {\rm{GR}}{{\rm{U}}^v}\ \left( {{\boldsymbol{v}}_i^{k^{\prime}},{\rm{\ }}{\boldsymbol{v}}_i^{k - 1}} \right)\end{equation*}$$
3. Graph update. The sum of the set of node feature vectors is concatenated with the graph feature vector ${{\boldsymbol{u}}^{k - 1}}$ of the previous layer as the input, which is fed into a nonlinear transformation ${\rm{ML}}{{\rm{P}}^u}$ to calculate the intermediate graph feature vector ${{\boldsymbol{u}}^{k^{\prime}}}$. Then, the graph feature vector ${{\boldsymbol{u}}^k}$ is updated using ${\rm{GR}}{{\rm{U}}^u}$.
  (21) $$\begin{equation*}{{\boldsymbol{u}}^{k^{\prime}}} = {\rm{ML}}{{\rm{P}}^u}\ \left( {\left[ {\mathop \sum \limits_{i\ = {\rm{\ }}1}^{{N_v}} {\boldsymbol{v}}_i^k;{{\boldsymbol{u}}^{k - 1}}} \right]} \right)\end{equation*}$$
  (22) $$\begin{equation*}{{\boldsymbol{u}}^k} = {\rm{GR}}{{\rm{U}}^u}\ \left( {{{\boldsymbol{u}}^{k^{\prime}}},{\rm{\ }}{{\boldsymbol{u}}^{k - 1}}} \right)\end{equation*}$$

#### Multilayer perceptron classifier

In GraphBind, four graph feature vectors are obtained from the four GNN-blocks. We concatenate them as the final representation of the target residue due to the following reasons: (1) the performance of deep GNNs may degrade due to the locally diverse graph structures (40); (2) the back-propagation path of each layer becomes shorter, which can accelerate the convergence of the model. Then, the concatenated graph feature vectors are fed into a multilayer perceptron classifier (CLF) to obtain the probability of being a binding residue${\rm{\ }}\hat{y}$:

(23) $$\begin{equation*}\hat{y} = {\rm{\ softmax}}\left( {{{\boldsymbol{W}}_2}{\rm{max}}\left( {0,{{\boldsymbol{W}}_1}[{{{\boldsymbol{u}}^1}; \ldots ;\ {{\boldsymbol{u}}^K}}] + {{\boldsymbol{b}}_1}}\right) + {{\boldsymbol{b}}_2}}\right)\end{equation*}$$

where ${\rm{softmax\ }}( {{x_i}} ) = {e^{{x_i}}}/( {1 + \mathop \sum \limits_j {e^{{x_j}}}} )$,$K=4$, ${{\boldsymbol{u}}^k} \in {\mathbb{R}^{{D_u}}}$, $k= [ {1,\ldots,K} ]$, ${{\boldsymbol{W}}_1} \in {\mathbb{R}^{256\times( {K{D_u}} )}}$, ${{\boldsymbol{b}}_1} \in {\mathbb{R}^{256}}$, ${{\boldsymbol{W}}_2} \in {\mathbb{R}^{2\times256}}$ and ${{\boldsymbol{b}}_2} \in {\mathbb{R}^2}$.

Instead of using a default threshold 0.5 to binarize the continuous value $\hat{y}$ into binding or non-binding residue class, the optimal threshold is determined by maximizing MCC on the validation set for each benchmark datasets.

### Baseline and state-of-the-art methods

In this study, we compare GraphBind with two types of methods. (1) A geometric-agnostic baseline method, biLSTMClf, is designed to demonstrate the advantages of geometric knowledge and the HGNN in GraphBind. (2) State-of-the-art methods for nucleic-acid-binding residue prediction are compared to demonstrate the effectiveness of GraphBind.

#### A geometric-agnostic baseline method biLSTMClf

**Figure 4.**
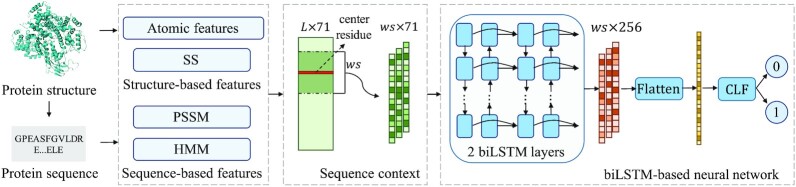
Pipeline of the geometric-agnostic baseline method biLSTMClf. The same residue features as GraphBind are extracted from the protein sequences and structures. A symmetrical sliding window is used to extract the sequence contexts of residues. We design the biLSTM-based neural network, which consists of two biLSTM layers and a multilayer perceptron classifier (CLF), to distinguish binding residues from non-binding residues.

As shown in Figure 4, biLSTMClf uses the same residue features derived from protein sequences and structures as GraphBind to represent a protein as an $L \times 71$ matrix, where $L$ stands for the length of a sequence. A symmetrical sliding window (6,41–42) is used to capture the sequence contexts instead of the structural contexts for target residues. Thus, a target residue is represented as a $ws \times 71$ matrix, where $ws$ stands for the size of the sliding window. After obtaining the initial features for target residues, a two-layer bidirectional long short-term memory network (biLSTM) is employed to extract the latent representations of residues. Then, a multilayer perceptron classifier (CLF), which is also used as the classifier in GraphBind, is used to predict the binding probability. biLSTMClf is a geometric-agnostic baseline and it is applied to evaluate whether the geometric knowledge is necessary for binding residue prediction and if GraphBind can learn informative latent embeddings from the geometric knowledge.

#### State-of-the-art methods

To demonstrate the effectiveness of GraphBind, we compare it with eight state-of-the-art methods, including deep-learning-based methods, shallow-machine-learning-based methods, template-based methods and consensus methods:

1. TargetDNA: a sequence-based method for DNA-binding residue prediction. It takes the evolutionary information and predicted secondary structure profiles as input and uses multiple SVMs with boosting as the classifier (6).
2. TargetS: a sequence-based method for ligand-binding residue prediction that takes the evolutionary information, predicted secondary structure profiles and ligand-specific propensity as input and employs the AdaBoost algorithm as the classifier (7).
3. NucBind: a consensus method for nucleic-acid-binding residue prediction. NucBind fuses a sequence-based method SVMnuc and a consensus method COACH-D (42,43).
4. DNAPred: a sequence-based method for DNA-binding residue prediction. DNAPred proposes a two stage imbalanced learning algorithm to decrease the impact of data imbalance problem with an ensemble technique (44).
5. RNABindRPlus: a consensus method for RNA-binding residue prediction. RNABindRPlus combines outputs from a sequence homology-based method with those from a SVM classifier (45).
6. NucleicNet: a structure-based deep learning method to predict RNA-binding preference on protein surfaces. NucleicNet analyzes physicochemical properties of grid points on protein surface and takes a deep residual network as the classifier. The binding score of a residue is averaged by scores of its 30 nearest grid points (13).
7. aaRNA: a sequence- and structure-based artificial neural network classifier for RNA-binding residue prediction. aaRNA employs a structural descriptor Laplacian norm to measures surface convexity/concavity over different length scales (12).
8. DNABind: a consensus method for DNA-binding residue prediction. DNABind integrates a sequence-based SVM classifier, a structure-based SVM classifier and a template-based method. DNABind extracts four topological features including degree, closeness, betweenness, and clustering coefficient to represent the geometric knowledge (46).

### Evaluation measurement

To assess the performance of GraphBind and other methods, we report the following five metrics. The four metrics for binary outputs, recall (Rec), precision (Pre), F1-score (F1), and Matthews correlation coefficient (MCC), are calculated as follows:

(24) $$\begin{equation*}{\rm Rec}\ = {\rm{\ }}\frac{{{\rm TP}}}{{{\rm TP} + {\rm FN}}}\end{equation*}$$

(25) $$\begin{equation*}{\rm Pre}\ = {\rm{\ }}\frac{{{\rm TP}}}{{{\rm TP} + {\rm FP}}}\end{equation*}$$

(26) $$\begin{equation*}{\rm F1}{\rm{\ }} = \frac{{2\cdot{\rm Rec}\cdot{\rm Pre}}}{{{\rm Rec} + {\rm Pre}}}\ \end{equation*}$$

(27) $$\begin{equation*}{\rm MCC} = \frac{{{\rm TP} \cdot {\rm TN} - {\rm FN} \cdot {\rm FP}}}{{\sqrt {\left( {{\rm TP} + {\rm FN}} \right)\left( {{\rm TP} + {\rm FP}} \right)\left( {{\rm TN} + {\rm FN}} \right)\left( {{\rm TN} + {\rm FP}} \right)} }}\ \end{equation*}$$

where TP, FP, TN and FN are abbreviations for true positives (number of correctly predicted samples as binding residues), false positives (number of incorrectly predicted samples as binding residues), true negatives (number of correctly predicted samples as non-binding residues) and false negatives (number of incorrectly predicted samples as non-binding residues). Recall measures the proportion of true binding residues that are correctly predicted as binding residues. Precision measures the proportion of the true binding residues in the predicted binding residues. F1 and MCC are calculated from multiple indicators and are objective metrics when the positive-negative sample ratio is not balanced.

In addition, we report the area under the receiver operator characteristic (ROC) curve (AUC) to assess the prediction score. ROC is a graphical plot of the true positives ratio against the false positives ratio over the entire range of different thresholds for the probability. Of the five metrics, F1, MCC and AUC are overall metrics, especially when the test set is imbalanced. All the reported metrics are averaged values of 10 repeated running of the methods.

### Significance test

Significance tests are performed to investigate if the improvement of MCCs and AUCs are due to a noisy estimate of model performance. Similar to the procedure used in previous studies (4,42), we randomly sample 70% of the test set and calculate the MCCs and AUCs of the best-performing method and other methods, which is repeated 10 times. The Anderson-Darling test is used to evaluate if the measurements are normal. If the measurement is normal, the paired t-test is used to calculate significance of the measurement. otherwise, the Wilcoxon rank sum test is applied. If the obtained *P*-value <0.05, the difference between a given pair of methods is considered statistically significant.

### Experimental settings

Twenty percent of the proteins from the original training set in Table 1 are used to construct the validation set ${V_{val}}$ and the remaining protein chains are used to construct the training set ${V_{tr}}$. We also use CD-HIT to ensure that the sequence similarity between the validation set and the training set is less than 30%. During the training process, grid search is used to find optimal hyperparameters.

We employ the Adam optimizer with ${\beta _1} = 0.9$, ${\beta _2} = 0.999$, ${\rm{\varepsilon}} = {10^{ - 8}}$ and learning rate is $5\times {10^{ - 5}}$ for model optimization on the cross-entropy loss as:

(28) $$\begin{equation*}{\rm{Loss\ }} = \mathop \sum \limits_{{v_i} \in {V_{tr}}} \left( {{y_i}ln\widehat {{y_i}} + \left( {1 - {y_i}} \right)ln\left( {1 - \widehat {{y_i}}} \right)} \right)\ \end{equation*}$$

where ${y_i}$ is the label of a residue and $\widehat {{y_i}}$ is the probability corresponding to ${y_i}$.

Dropout (47) is applied to each MLP module with a rate of ${P_{drop}} = \ 0.5$ to avoid overfitting. To accelerate convergence and improve generalization performance, batch normalization (48) is employed on every convolution layer in MLP.

## RESULTS

In this section, we first conduct ablation studies to investigate different settings on the performance of GraphBind. Then, we compare GraphBind with the geometric-agnostic baseline and state-of-the-art methods on the nucleic-acid-binding benchmark datasets to demonstrate the advantages of the proposed structural-context-based graph representations and the HGNN. Moreover, we investigate the contributions of different features, the impact of data augmentation with transferring binding annotations, and how they impact GraphBind when using predicted structures from sequences.

### Ablation studies on GraphBind

To investigate the contributions of different settings of GraphBind, we conduct ablation studies on GraphBind with different settings on the validation set from DNA-573_Train. These results are given in Table 2.

**Table 2.** Ablation studies on GraphBind with different settings^a^

	Pos^b^	PE^c^	r_g_^d^	r_v_^e^	EU^f^	A^g^	GRU^h^	N_L_^i^	D^j^	Rec	Pre	F1	MCC	AUC
Base	C	T	20	10	T	S	T	4	128	0.676	0.537	**0.598**	**0.558**	**0.926**
(A)	SC									0.593	0.591	0.592	0.552	0.922
	CA									0.633	0.537	0.581	0.538	0.921
(B)		F								0.650	0.528	0.583	0.540	0.920
(C)			15							0.634	0.551	0.589	0.548	0.919
			25							0.656	0.540	0.593	0.551	0.923
			30							0.580	**0.594**	0.587	0.547	0.913
(D)				5						0.622	0.472	0.537	0.490	0.910
				13						0.663	0.540	0.595	0.555	0.923
(E)					F					0.570	0.483	0.523	0.474	0.899
(F)						M				0.561	0.407	0.472	0.418	0.875
(G)								2		0.630	0.524	0.573	0.529	0.914
								3		0.647	0.551	0.595	0.554	0.925
								5		0.647	0.545	0.592	0.550	0.925
								6		**0.688**	0.522	0.586	0.545	0.924
							F	2		0.670	0.523	0.587	0.546	0.925
							F	4		0.637	0.541	0.585	0.543	0.922
							F	6		0.669	0.504	0.575	0.533	0.922
(H)									64	0.593	0.527	0.558	0.513	0.910

^a^Only different settings are given and other settings (empty values) are the same as the base model. These metrics are calculated on the validation set of DNA-573_Train and the highest values are bolded.

^b^Pseudo-position of a residue: C, SC and CA stand for the centroid of residue, the centroid of residue side-chain and the position of alpha-C atom, respectively.

^c^Use the relative distance from every node to the sphere center as position embeddings of nodes (T) or not (F).

^d^Radius of the structural context: it defines the nodes belonging to a graph of a residue, and its unit is Å.

^e^The threshold of adjacent matrix: it binarizes a distance matrix to the adjacent matrix to define the adjacent edges belonging to a node, and its unit is Å.

^f^Use the edge feature vectors (T) or not (F).

^g^The aggregation operation in the node update module and the graph update module. S and M stand for sum and max operation, respectively.

^h^Use GRU (T) or not (F). If GRU is not used, the output $e_{ij}^k$,${\rm{\ }}v_i^k$,$\ {u^k}$ equal the intermediate output $e_{ij}^{k^{\prime}}$, $v_i^{k^{\prime}}$, ${U^{k^{\prime}}}$, respectively.

^i^The number of GNN-blocks.

^j^ *D*_e_, *D*_v_ and *D*_u_ stand for the dimension of encoded edge feature vectors, the dimension of encoded node feature vectors and the dimension of encoded graph feature vectors, respectively. We set *D*_e_$ = {\rm{\ }}$*D*_v_$ = {\rm{\ }}$*D*_u_.

As shown in Table 2, experiments A–D evaluate the contributions of different settings for graph construction. As shown in the experiment A, pseudo-positions denoted by the centroid of residues yields higher performance than denoted by the alpha-C atoms, and achieves similar results to be denoted by the centroid of the residue side-chains. The results demonstrate that centroid of residues or residue side-chains are more correlated to binding sites than sole backbone alpha-C atoms. The experiment B shows that it is beneficial to take the relative distance between each node and the sphere center as the position embedding, since the position embedding can be used to distinguish nodes when updating the graph feature vector. As shown in the experiment C and D, a smaller radius of the structural context and fewer edges limit the receptive field of the network, resulting in a worse performance. However, a larger radius for the structural context and more edges also bring no benefit to the performance but take longer training time.

We also test different network components for the HGNN in GraphBind. In the experiment E, the edge feature vectors are ignored, and the Eqs. (15) and (19) are replaced by Eqs. (29) and (30), respectively.

(29) $$\begin{equation*}{\boldsymbol{v}}_i^{enc} = {\rm{MLP}}_{enc}^v\ \left( {\left[ {{\rm{\ }}{\boldsymbol{v}}_i^{raw};{\rm{\ }}\mathop \sum \limits_{j \in N\left( {{v_i}} \right)} {\boldsymbol{v}}_j^{raw}} \right]} \right)\end{equation*}$$

(30) $$\begin{equation*}{\boldsymbol{v}}_i^{k^{\prime}} = {\rm{ML}}{{\rm{P}}^v}\ \left( {\left[ {{\rm{\ }}{\boldsymbol{v}}_i^{k - 1};{\rm{\ }}\mathop \sum \limits_{j \in N\left( {{v_i}} \right)} {\rm{\ }}{\boldsymbol{v}}_j^{k - 1};{{\boldsymbol{u}}^{k - 1}}} \right]} \right)\end{equation*}$$

The decreasing performance of the experiment E demonstrates the importance of integrating edge feature vectors into the node update module and the importance of the geometric knowledge. The experiment F applies the max aggregation instead of sum aggregation, leading to a lower performance. It is probably because the max pooling operation only records the maximum value and loses the information of other nodes. As shown in the experiment G, we investigate the impact of the number of GNN-Blocks and stacking these GNN-Blocks with or without GRU operation. If GRU is not used, ${\rm{GR}}{{\rm{U}}^e}$, ${\rm{GR}}{{\rm{U}}^v}$ and ${\rm{GR}}{{\rm{U}}^u}$ are removed, and the outputs ${\boldsymbol{e}}_{ij}^k$,${\rm{\ }}{\boldsymbol{v}}_i^k$,$\ {{\boldsymbol{u}}^k}$ are set as the intermediate outputs ${\boldsymbol{e}}_{ij}^{k^{\prime}}$, ${\boldsymbol{v}}_i^{k^{\prime}}$, ${{\boldsymbol{u}}^{k^{\prime}}}$, respectively. The results show stacking only two GNN-blocks leads to performance degradation, since the receptive field of stacking fewer GNNs is limited. Adding more GNN-Blocks without GRU also leads to a worse performance. The result demonstrates that deeper GNN can benefit from GRU because it takes advantage of all blocks’ information. The experiment H shows that setting latent representation of the size of 128 for edges, nodes and graphs can extract more discriminate features and yields better performance.

### GraphBind is superior to geometric-agnostic biLSTMClf

**Figure 5.**
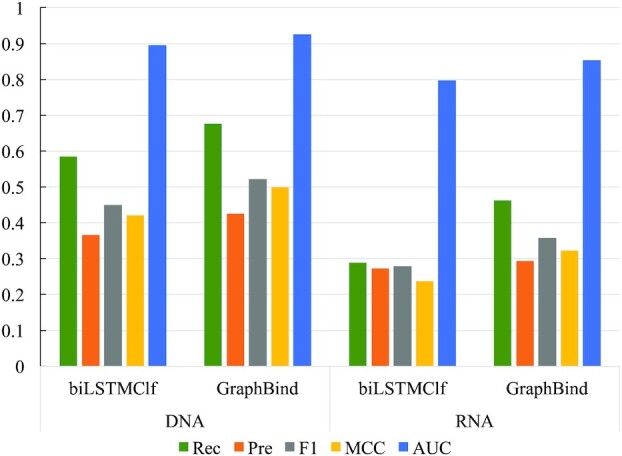
Performance comparison between biLSTMClf and GraphBind on nucleic-acid-binding test sets.

We benchmark GraphBind against the geometric-agnostic baseline method biLSTMClf. These two methods share the same training sets, validation sets and test sets. Performance comparison between biLSTMClf and GraphBind is shown in Figure 5 (see Supplementary Table S2 for details). GraphBind yields higher F1-score, MCC and AUC, which are 0.072(0.078), 0.079 (0.084) and 0.031(0.056) higher than those of biLSTMClf on DNA(RNA)-binding benchmark sets, respectively. Two observations can be drawn from the comparison. First, geometric knowledge is necessary for DNA/RNA-binding residue recognition task. Second, GraphBind is more effective than biLSTMClf for learning latent embeddings of local patterns around target residues, since GraphBind can abstract the patterns of local structures in an end-to-end way from both geometric knowledge and bio-physicochemical characteristics.

### Comparison with state-of-the-art methods on benchmark sets

For the purely sequence-based methods (i.e. TargetDNA, TargetS, DNAPred and RNABindRPlus) we upload the protein sequences of the test sets to their webservers. For the methods with structures as the input, we upload the PDB files (49) of the test sets to their webservers or standalone softwares.

**Figure 6.**
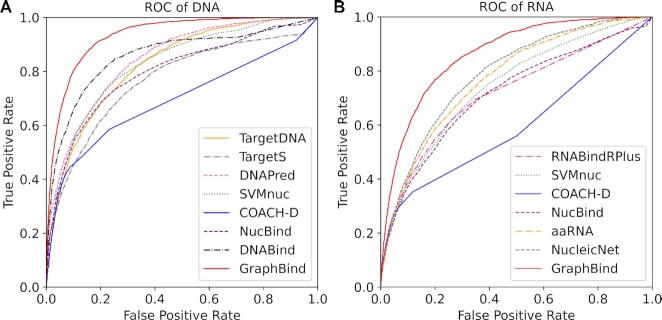
The ROC curves for GraphBind and state-of-art methods on DNA-129_Test(A) and RNA-117_Test(B).

Performance comparison of GraphBind with state-of-the-art methods on nucleic-acid-binding test sets are reported in Table 3 and the ROC curves are provided in Figure 6. As shown in Table 3, GraphBind yields a better performance than state-of-the-art methods. The F1-score, MCC and AUC of GraphBind are 0.082(0.097), 0.088(0.106) and 0.069(0.066) higher than the second highest values on DNA(RNA)-binding test set, they are a relative increase of 18.6%(37.2%), 21.4%(49.1%), 8% (8.4%), respectively. The MCCs of the structure-based methods (DNABind, aaRNA, NucleicNet and GraphBind) are generally higher than those of sequence-based methods (TargetDNA, DNAPred, TargetS, RNABindRPlus and SVMnuc), indicating the importance of structural information. The lower AUCs of the template-based method COACH-D are probably because the similarities between the templates and the queries are not high enough, leading to many zero scores for the queries (42). The superiority of GraphBind over DNABind and aaRNA proves that the structural-context-based graph representation is more suitable for representing the local structural information of residues than the hand-crafted structural descriptors for recognizing the binding residues. In addition, the superiority of GraphBind over NucleicNet demonstrates that the HGNN in GraphBind can capture more important geometric and bio-physicochemical characteristics from graph representation than those captured with CNNs from 2D image representation in NucleicNet. Furthermore, significance tests are performed between GraphBind and other methods, which shows that the improvement on MCCs and AUCs are statistically significant. ROC curves shown in Figure 6A and B also verify the effectiveness of GraphBind. In addition, we calculate the MCC of each protein chain independently and draw the distribution of MCCs for the second-best DNA-binding predictor DNABind, the second-best RNA-binding predictor NucleicNet and GraphBind in Supplementary Figure S1, which also verifies the performance of GraphBind.

**Table 3.** Performance comparison of GraphBind with state-of-the-art methods on nucleic-acid-binding test sets^a^

Dataset	Method	Rec	Pre	F1	MCC	*P*-values of MCC	AUC	*P*-values of AUC
DNA-129_Test	TargetDNA^b^	0.417	0.280	0.335	0.291	1.45 $ \times$ 10^−11^	0.825	1.64 $ \times$ 10^−11^
	TargetS^c^	0.239	0.370	0.291	0.262	4.85 $ \times$ 10^−12^	N/A	N/A
	DNAPred^d^	0.396	0.353	0.373	0.332	7.09 $ \times$ 10^−12^	0.845	2.14 $ \times$ 10^−11^
	SVMnuc^e^	0.316	0.371	0.341	0.304	1.89 $ \times$ 10^−12^	0.812	1.98 $ \times$ 10^−11^
	COACH-D^e^	0.324	0.360	0.341	0.302	1.99 $ \times$ 10^−13^	0.761	8.60 $ \times$ 10^−16^
	NucBind^e^	0.323	0.373	0.346	0.309	3.72 $ \times$ 10^−13^	0.797	6.38 $ \times$ 10^−11^
	DNABind^f^	0.601	0.346	0.440	0.411	1.04 $ \times$ 10^−8^	0.858	1.57 $ \times$ 10^−11^
	GraphBind	**0.676** $ \pm$**0.027**	**0.425** $ \pm$**0.017**	**0.522** $ \pm$**0.005**	**0.499** $ \pm$**0.004**	N/A	**0.927** $ \pm$**0.006**	N/A
RNA-117_Test	RNABindRPlus^g^	0.273	0.227	0.248	0.202	2.96 $ \times$ 10^−10^	0.717	8.42 $ \times$ 10^−13^
	SVMnuc	0.231	0.240	0.235	0.192	7.21 $ \times$ 10^−11^	0.729	9.28 $ \times$ 10^−13^
	COACH-D	0.221	0.252	0.235	0.195	3.99 $ \times$ 10^−11^	0.663	1.14 $ \times$ 10^−12^
	NucBind	0.231	0.235	0.233	0.189	8.24 $ \times$ 10^−12^	0.715	1.29 $ \times$ 10^−11^
	aaRNA^h^	**0.484**	0.166	0.247	0.214	5.61 $ \times$ 10^−11^	0.771	2.45 $ \times$ 10^−12^
	NucleicNet^i^	0.371	0.201	0.261	0.216	4.64 $ \times$ 10^−10^	0.788	1.03 $ \times$ 10^−10^
	GraphBind	0.463 $ \pm$0.036	**0.294** $ \pm$**0.017**	**0.358** $ \pm$**0.008**	**0.322** $ \pm$**0.008**	N/A	**0.854** $ \pm$**0.006**	N/A

^a^We report the averages and standard deviations after having performed the experiments ten times.

^b^Results are computed using the TargetDNA server at http://csbio.njust.edu.cn/bioinf/TargetDNA/.

^c^Results are computed using the TargetS server at http://www.csbio.sjtu.edu.cn/bioinf/TargetS/.

^d^Results are computed using the DNAPred server at http://csbio.njust.edu.cn/bioinf/dnapred/

^e^Results are computed using the NucBind server at http://yanglab.nankai.edu.cn/NucBind/.

^f^Results are computed using the DNABind server at http://mleg.cse.sc.edu/DNABind/.

^g^Results are computed using the RNABindRPlus server at http://ailab-projects2.ist.psu.edu/RNABindRPlus/.

^h^Results are computed using the aaRNA server at http://sysimm.ifrec.osaka-u.ac.jp/aarna/.

^i^Results are computed using the standalone program at https://github.com/NucleicNet/NucleicNet.

### Case studies

In this section, we visualize two cases from the test sets predicted by GraphBind and the second-best methods DNABind and NucleicNet for DNA-binding proteins and RNA-binding proteins, respectively. We select two cases that have MCCs close to the overall MCCs (shown in Table 3) on the DNA-129_Test and RNA-117_Test, respectively. One is the DNA-binding protein 5WX9_A, and the other is the RNA-binding protein 5Z9W_A.

**Figure 7.**
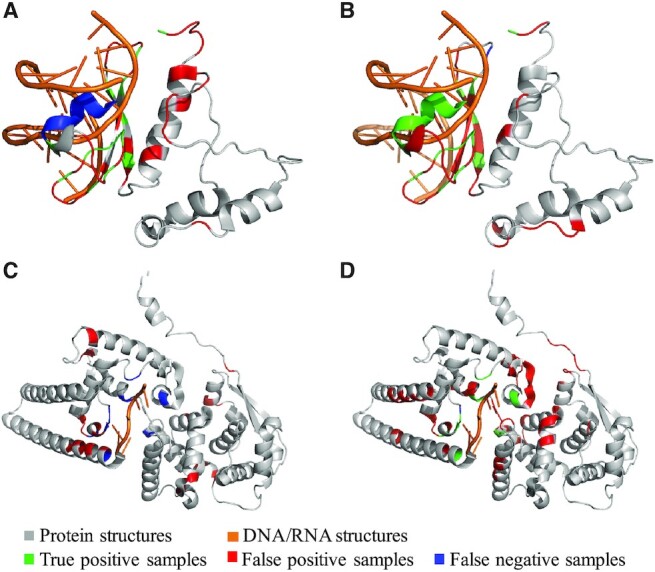
Visualization of two cases predicted by GraphBind and the second-best methods. For the first protein chain 5WX9_A from DNA-129_Test, the results predicted by DNABind(**A**) and GraphBind(**B**) are shown. For the second protein chain 5Z9W_A from RNA-117_Test, the results predicted by NucleicNet(**C**) and GraphBind(**D**) are shown.

The DNA-binding protein 5WX9_A has 131 residues, and 21 of them are binding residues (Figure 7A and B). GraphBind currently predicts 20 true binding residues and 32 false positive residues. For this protein, GraphBind achieves Rec = 0.952, Pre = 0.385, F1 = 0.548, MCC = 0.496 and AUC = 0.945. On this case, DNABind predicts only 14 true binding residues and 32 false positive residues, achieving Rec = 0.667, Pre = 0.304, F1 = 0.418, MCC = 0.289 and AUC = 0.806.

The RNA-binding protein 5Z9W_A has 388 residues, 11 of them are binding residues (Figure 7C and D). For this protein, GraphBind predicts 10 true binding residues and only one true binding residue is missed, yielding a performance with Rec = 0.909, Pre = 0.154, F1 = 0.263, MCC = 0.339 and AUC = 0.938. However, NucleicNet predicts no binding residue in 5Z9W_A. All of the 11 true binding residues are incorrectly predicted as non-binding residues, yielding a Rec = 0.000, Pre = 0.000, F1 = 0.000, MCC = –0.041 and AUC = 0.760.

### Feature importance analysis

**Figure 8.**
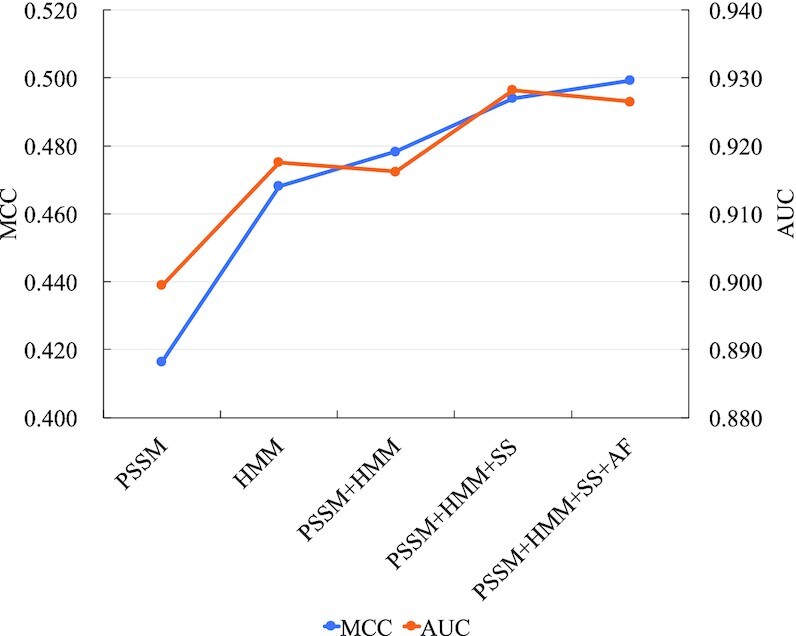
Performance of GraphBind with the different feature combinations of residue features on DNA-129_Test.

As mentioned above, we extract the atomic features of residues (AF) and secondary structure profiles (SS) from protein structures, as well as PSSM and HMM profiles from protein sequences. In this section, we investigate the impacts of different feature combinations for GraphBind. On DNA-129_Test, we evaluate GraphBind with the following 5 feature combinations: (i) PSSM, (ii) HMM, (iii) PSSM+HMM, (iv) PSSM+HMM+SS and (v) PSSM+HMM+SS+AF. Figure 8 illustrates the MCC and AUC against different feature combinations, and the detailed metrics are reported in Supplementary Table S3.

As shown in Figure 8, when looking at the single feature, HMM is more discriminate against PSSM. When combining HMM and PSSM, GraphBind yields improvement in the MCC, which is a more objective metric than AUC for imbalanced data. Integrating secondary structure features further improves the performance of GraphBind. Finally, GraphBind with the combination of all these features yields the highest MCC, indicating that these four kinds of features are complementary.

### The impact of data augmentation with transferring binding annotations

**Figure 9.**
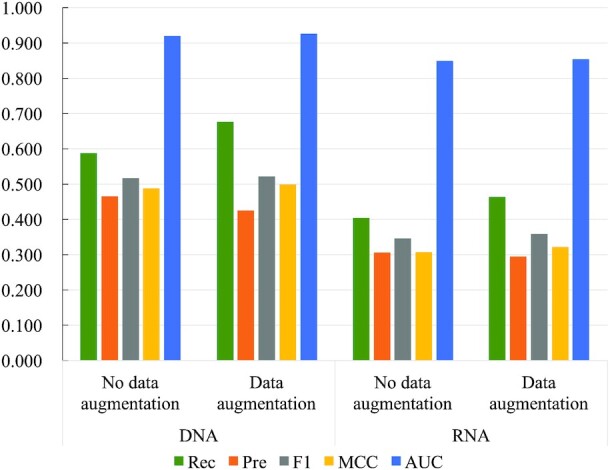
Performance comparison of GraphBind trained on the nucleic-acid-binding training sets with or without data augmentation by transferring binding annotations.

In this study, we transfer binding annotations from similar proteins as a data augmentation method to increase the number of binding residues in the training sets. After transferring the annotations, the numbers of DNA- and RNA-binding residues in the training sets are expanded by 30.7% and 24.3%, respectively. We compare the performance of GraphBind trained on the training sets with and without data augmentation. The results on independent test sets are shown in Figure 9 (see Supplementary Table S4 for more details). For both DNA- and RNA-binding test sets, the higher recalls of GraphBind with data augmentation indicate that more true binding residues are identified. It is meaningful because DNA/RNA-binding residue prediction suffers from data imbalance and the majority of the training samples are non-binding residues. The results confirm the benefit of data augmentation.

### The impact of predicted protein structures on GraphBind

GraphBind is designed for constructing graphs and making predictions based on experimental protein structures. To test if GraphBind can be applied on a much larger population of proteins without experimental structures, we evaluate the performance of GraphBind with protein structures predicted by MODELLER (50) from protein sequences. We employ TM-align (31) to calculate the similarity between predicted structures and experimental structures in PDB (49). As shown in Supplementary Table S5, the predicted structures have a negative impact on the prediction performance of GraphBind. There are two main reasons. (i) The graphs constructed from structural contexts are directly derived from the positions of residues in protein structures, and those residues that are highly deviated from the experimental structure are no longer included in the structural contexts, leading to a negative impact in the constructed graphs. (ii) The adjacent matrix, position embeddings of nodes and raw edge feature vectors in the HGNN are also based on the position relationship of the residues.

We further compare GraphBind with sequence-based methods on the subsets consisting of predicted protein structures with the TM-scores >0.5 (Table 4). TM-scores >0.5 indicates a certain degree of similarity between experimental structures and predicted structures (51). As shown in Table 4, the recalls of GraphBind are higher than these sequence-based methods, which indicates GraphBind is preferred to predicting more residues as binding residues to improve the coverage of true binding residues when protein structures are changed.

**Table 4.** Comparison of GraphBind with the sequence-based methods on the subsets consisting of predicted protein structures with TM-scores >0.5 in the nucleic-acid-binding test sets^a^

Type	*N*_protein_^b^	Method	Rec	Pre	F1	MCC	AUC
DNA	71	TargetDNA	0.433	0.335	0.378	0.332	0.839
		TargetS	0.278	**0.451**	0.344	0.320	N/A
		DNAPred	0.423	0.433	**0.428**	**0.389**	**0.859**
		SVMnuc	0.320	0.408	0.358	0.323	0.796
		GraphBind	**0.500** $ \pm$**0.032**	0.346 $ \pm$0.016	0.408 $ \pm$0.007	0.367 $ \pm$0.008	0.838 $ \pm$0.012
RNA	44	RNABindRPlus	0.314	**0.307**	**0.310**	**0.265**	0.770
		SVMnuc	0.269	0.305	0.286	0.243	0.752
		GraphBind	**0.361** $ \pm$**0.036**	0.249 $ \pm$0.013	0.293 $ \pm$0.010	0.244 $ \pm$0.011	**0.795** $ \pm$**0.010**

^a^We report the averages and standard deviations after having performed the experiments ten times.

^b^The number of proteins with TM-scores >0.5 in the nucleic-acid-binding test sets.

In summary, although predicted structures degrade the performance of GraphBind, GraphBind also has a certain robustness when the structure transformation is not too large. This phenomenon inspires that we can construct graphs based on protein sequences to apply GraphBind on more proteins without experimental structures.

## DISCUSSION

In this section, the latent graph feature vectors are visualized to show the representation ability of GraphBind. In addition, GraphBind is trained and evaluated on other ligand-binding datasets to evaluate the generalization capability and practicality. Finally, we discuss the advantages and limitations of GraphBind.

### GraphBind learns effective latent graph feature vectors for residues

**Figure 10.**
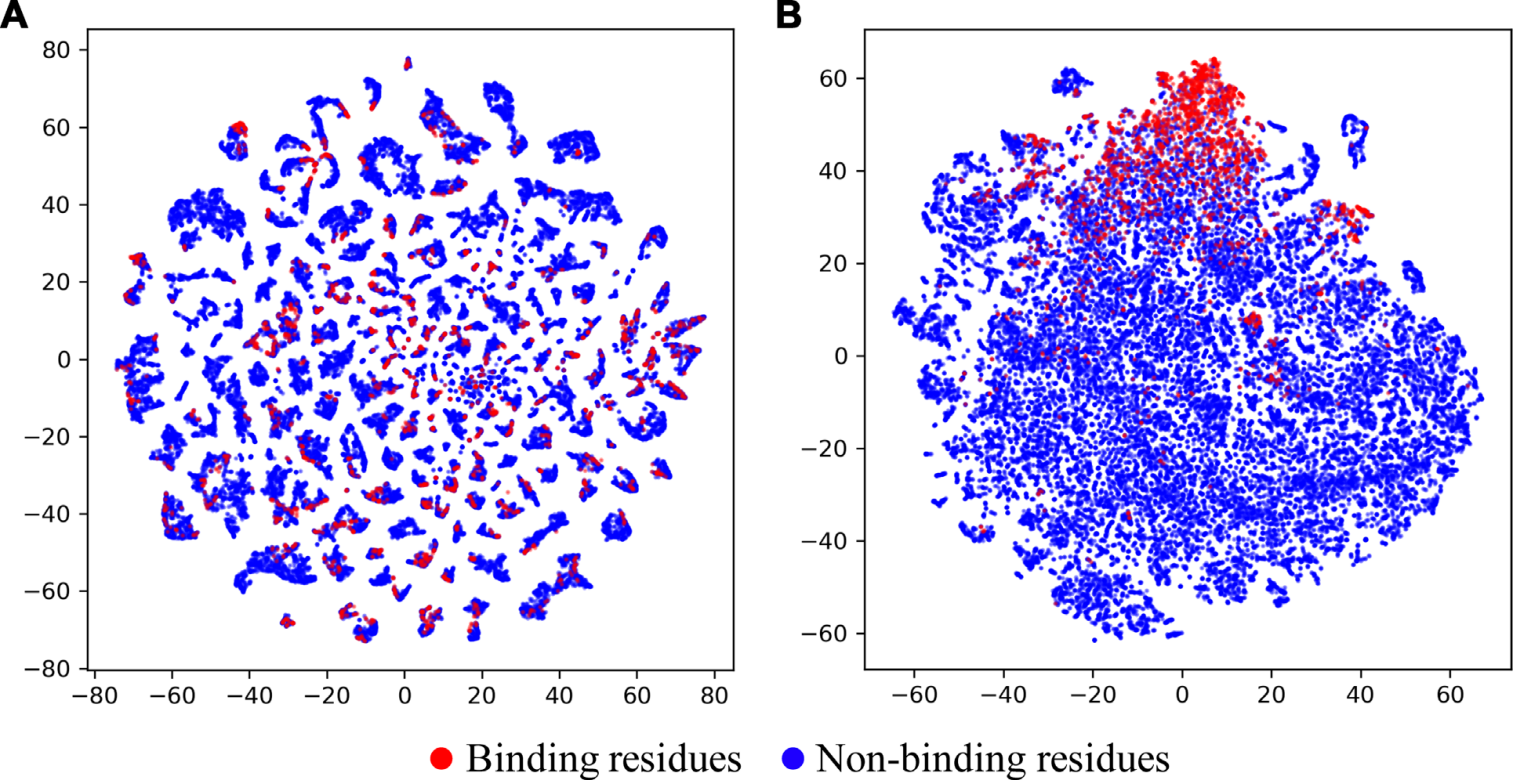
Visualization of the distribution of samples encoded by raw graph feature vectors (**A**) and latent graph feature vectors learned by GraphBind (**B**) on DNA-129_Test using t-SNE.

In this section, we employ t-SNE (52) to visualize the raw graph feature vectors and the latent graph feature vectors learned by GraphBind. For a target residue, the sum of the raw feature vectors of all nodes ${V^{raw}}$ in a graph serves as the raw graph feature vector, which has the size of 72. The latent graph feature vector learned by GraphBind with the size of 512 is the concatenation of embedded four graph feature vectors from four GNN-blocks. t-SNE is employed to project the high-dimensional feature vectors into the 2D space. Figure 10A and B illustrate the distribution of samples encoded by raw graph feature vectors and latent graph feature vectors on DNA-129_Test, respectively. As shown in Figure 10A, we can see that binding and non-binding residues overlap and are indistinguishable. Figure 10B shows that most binding residues are clustered together and separated from most non-binding residues. The results demonstrate that the latent graph representations learned by GraphBind greatly improves the discriminability of binding and non-binding residues.

### Extending GraphBind to other types of ligands

**Figure 11.**
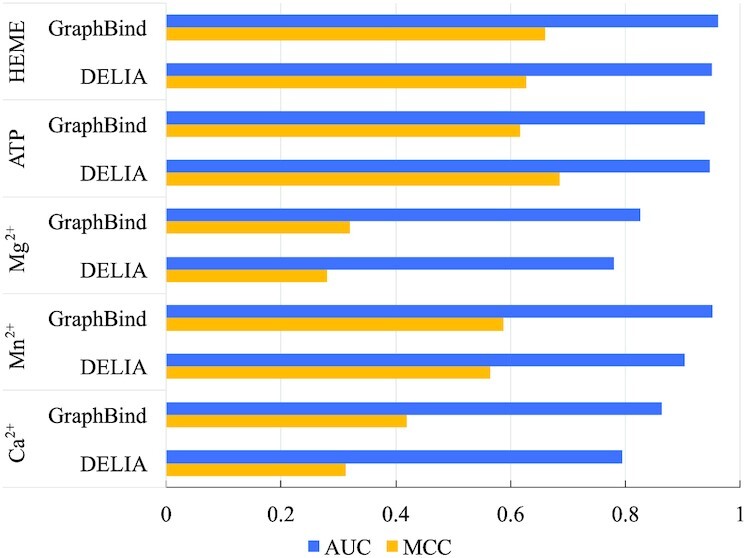
Performance comparison of GraphBind and DELIA on the five ligand-binding test sets.

We explore the applications of GraphBind in recognizing other types of ligand-binding residues. We compare GraphBind with TargetS (7), S-SITE (11), COACH (11), IonCom (53), ATPbind (54) and DELIA (19) on the five benchmark ligand sets collected from ATPbind (54) and DELIA (19), including three metal ions (i.e. Ca^2+^, Mn^2+^ and Mg^2+^) and two biologically relevant molecules (i.e. ATP and HEME). The details of the five benchmark sets are described in Supplementary Section S1 and Supplementary Table S6. They are selected for generalization test since the amount of binding residues of these ligands is large enough for our deep models. We follow the same training and evaluation protocol on these five types of ligands as stated in previous sections. Hyperparameters are adjusted on each ligand-specific validation set. The performance comparison of GraphBind with the six state-of-the-art methods are reported in Supplementary Table S7. The MCCs and AUCs of GraphBind and the state-of-the-art DELIA are illustrated in Figure 11. The results show that GraphBind yields an improvement of 0.023–0.107 on MCC and 0.011–0.068 on AUC on Ca^2+^, Mn^2+^, Mg^2+^ and HEME compared to the second-best DELIA. The results suggest that the graph constructed from protein structural context is more powerful and suitable in representing structure information than the 2D distance matrix, and GraphBind is also effective in predicting ligand-binding residues.

### Ligand-general GraphBind-G transferred from ligand-specific GraphBind still achieves a promising performance

GraphBind is a ligand-specific method which trains a model p1er ligand to learn ligand-specific binding patterns. Thus, GraphBind is limited to predict binding residues for those ligands with small number of verified binding residues. Differently, ligand-general methods train models on pooled binding residues from multiple ligands, so they learn the common patterns of a large types of ligands and are able to predict binding residues for unseen ligands but cannot predict which ligand the residue would bind to.

Here, we train a ligand-general model, GraphBind-G, with the same architecture as GraphBind.We compare the GraphBind-G with another ligand-general method, P2Rank (55). To make a fair comparison, we train and evaluate the ligand-general GraphBind-G on the ligand-general benchmark set from P2Rank. This benchmark set consists of a training set CHEN11, a validation set JOINED and a test set COACH420. The training set CHEN11 contains binding sites between 476 ligands and 251 proteins, and the test set COACH420 consists of binding sites between 420 proteins and a variety of drug targets and ligands. GraphBind is a residue-centric method. However, no ligand-binding residue annotations are given in this benchmark set. According to P2Rank, we define a ligand-binding residue with a distance less than 5.5 Å from the center of the mass of the ligand to the closest residue atom. For the pocket-centric P2Rank, we treat all residues in the predicted binding pockets as the predicted binding residues.

Performance comparison of the GraphBind-G and P2Rank on the COACH420 test set is summarized in Table 5. The higher recall and lower precision of P2Rank indicate that more positive binding residues are predicted with a higher false positive rate. It should be noted that P2Rank focuses on how to accurately predict the pocket positions of binding sites and assumes that a binding site may harbor a larger ligand, possibly leading to a higher false positive rate (55). The F1-score and MCC of GraphBind-G are 0.158 and 0.081 higher than those of P2Rank. The results indicate that the GNN-based GraphBind-G outperforms the random-forest-based P2Rank, demonstrating the advantages of GNNs over traditional machine learning methods and the validity of our method on ligand-general binding residue prediction. The general model of GraphBind-G is also available as an online service at the same website.

**Table 5.** Comparison of the performance of ligand-general GraphBind-G and P2Rank on the COACH420 test set^a^

Method	Rec	Pre	F1	MCC	AUC
P2Rank^b^	**0.888**	0.079	0.145	0.224	N/A
GraphBind-G	0.477 ±0.037	**0.223** **±0.013**	**0.303** **±0.007**	**0.305** **±0.008**	0.889 ±0.007

^a^We report the averages and standard deviations after having ran GraphBind-G ten times.

^b^Results are calculated based on the predictions from https://github.com/rdk/p2rank-datasets.

### The advantages of GraphBind

The superior performance of GraphBind over geometric-agnostic biLSTMClf demonstrate the importance of the geometric knowledge. Most of the compared methods first extract geometric and bio-physicochemical characteristics, then these features are fed into a supervised classifier for predicting binding residues (12,13). These methods separate the feature engineering and classification. For example, the deep-learning-based NucleicNet represents the structure as 2D image with physicochemical environment, which is further processed using CNNs for classifying binding residues (13). However, GraphBind is trained in an end-to-end way, it is able to refine the geometric and bio-physicochemical characteristics by taking the local structural context topology into account. In summary, the superior performance of GraphBind benefits from two aspects: (i) the graph representation based on structural context is suitable for representing the geometric and bio-physicochemical knowledge of target residue's local environment and (ii) the HGNN is an efficient algorithm to learn the high-level patterns for binding residue prediction.

### The limitations of GraphBind

Current GraphBind performs predictions upon protein structures. As shown in Table 4, taking predicted structures as inputs for GraphBind would reduce its performance, suggesting the structure quality matters the geometric knowledge, which is important for the HGNN. In the future work, we expect to figure out a new approach to build heterogeneous graphs through integrating protein primary sequences, which may be robust to the structure information alone. Another potential extension of current GraphBind is to add the module of predicting specific DNA/RNA interaction components, which would provide more useful clues for deeply understanding the interaction mechanisms (13).

## CONCLUSION

In this study, we propose GraphBind, protein structural context embedded rules learned by the hierarchical graph neural network (HGNN) for recognizing nucleic-acid-binding residues. Considering that nucleic-acid-binding residues are mainly determined by the local patterns of protein tertiary structures and bio-physicochemical environment, we first present a structural-context-based graph representation to represent the bio-physicochemical characteristics and geometric knowledge of residues and their varying number of the unordered neighbors, and it has the invariance of rotation and translation. Furthermore, the HGNN is proposed to learn the effective fixed-size latent representations from edge and node feature vectors of graphs. The results demonstrate the superiority of GraphBind on recognizing nucleic-acid-binding residues, and the generalization capability on identifying binding residues for multiply types of ligands and general ligands.

## DATA AVAILABILITY

The data and web server are freely available at http://www.csbio.sjtu.edu.cn/bioinf/GraphBind/, and the source code of GraphBind is available at http://www.csbio.sjtu.edu.cn/bioinf/GraphBind/sourcecode.html.

## Supplementary Material

gkab044_Supplemental_File
