# Supplementary material for: GraphBind: protein structural context embedded rules learned by hierarchical graph neural networks for recognizing nucleic-acid-binding residues
Source: Nucleic Acids Res. 2021 Feb 12;49(9):e51. doi: 10.1093/nar/gkab044 (PMC8136796; doi:10.1093/nar/gkab044)
Supplement: gkab044_Supplemental_File [file gkab044_supplemental_file.docx]

**GraphBind: protein structural context embedded rules learned by hierarchical graph neural networks for recognizing nucleic-acid-binding residues**

Ying Xia^1^, Chun-Qiu Xia^1^, Xiaoyong Pan^1,*^, and Hong-Bin Shen^1,2,*^

^1^ Institute of Image Processing and Pattern Recognition, Shanghai Jiao Tong University, and Key Laboratory of System Control and Information Processing, Ministry of Education of China, Shanghai, 200240, China

^2^ School of Life Sciences and Biotechnology, Shanghai Jiao Tong University, Shanghai, 200240, China

**SUPPLEMENTARY DATA**

**Supplementary sections**

- Section S1. Generalization test data of GraphBind model.

**Supplementary tables**

- Table S1. Nucleic acid-binding training sets without data augmentation.
- Table S2. Performance comparison with the baseline method on DNA- and RNA-binding test sets. We report the average after having performed each experiment ten times.
- Table S3. Performance of GraphBind with different combinations of residue features on DNA-129_test. We report the average after having performed each experiment ten times.
- Table S4. The performance of GraphBind trained on the nucleic acid-binding training sets without data augmentation. We report the average after having performed each experiment ten times.
- Table S5. The performance of GraphBind using the predicted protein structures from sequences. We report the average after having performed each experiment ten times.
- Table S6. Summary of the five benchmark datasets from DELIA.
- Table S7. Performance comparison of GraphBind with other state-of-the-art methods on test sets of metal ions (i.e. Ca^2+^, Mn^2+^ and Mg^2+^) and biologically relevant molecules (i.e. ATP and HEME). N/A means the metric cannot be measured since the webserver or software only outputs binary scores. We report the average after having performed each experiment ten times.

**Supplementary Figures**

- Figure S1. The distribution of predicted MCCs of each protein chain in the test set. Figures A, B, C and D corresponds to DNABind on DNA-129_Test, GraphBind on DNA-129_Test, NucleicNet on RNA-117_Test and GraphBind on RNA-117_Test, respectively.

**Supplementary sections**

Section S1. Generalization test data of GraphBind model.

The Ca^2+^-, Mn^2+^-, Mg^2+^- and HEME-binding protein datasets are download from DELIA(1). Protein chains released before January 6, 2016, are assigned into training sets, and the remaining chains are used as test sets. For each ligand, CD-HIT is applied to reduce the sequence similarity to 30% between any pair of the union set of the training set and the test set of this ligand. The ATP-binding protein training set ATP-388_Train and test set ATP-41_Test are downloaded from ATPbind(2). The sequence similarity between any pair of protein chains in the ATP dataset is lower than 40%. The details of the training and test sets for five small ligands are given in Supplementary Table S7.

**Supplementary tables**

Table S1. Nucleic acid-binding training sets without data augmentation.

| Type | Dataset | N_protein_^a^ | N_pos_^b^ | N_neg_^c^ | PNratio^d^ |
| --- | --- | --- | --- | --- | --- |
| DNA | DNA-573_Train | 573 | 11074 | 148809 | 0.074 |
| RNA | RNA-495_Train | 495 | 11756 | 125143 | 0.094 |

^a^ Number of proteins.

^b^ Number of binding residues.

^c^ Number of nonbinding residues.

^d^ PNratio = N_pos_/N_neg_.

Table S2. Performance comparison with the baseline method on DNA- and RNA-binding test sets. We report the average after having performed each experiment ten times.

| Dataset | Method | Rec | Pre | F1 | MCC | AUC |
| --- | --- | --- | --- | --- | --- | --- |
| DNA  (DNA-129_Test) | biLSTMClf | 0.585  $\pm$0.028 | 0.366  $\pm$0.015 | 0.450  $\pm$0.006 | 0.420  $\pm$0.006 | 0.896  $\pm$0.002 |
|  | GraphBind | **0.676**  $\boldsymbol{\pm}$**0.027** | **0.425**  $\boldsymbol{\pm}$**0.017** | **0.522**  $\boldsymbol{\pm}$**0.005** | **0.499**  $\boldsymbol{\pm}$**0.004** | **0.927**  $\boldsymbol{\pm}$**0.006** |
| RNA  (RNA-117_Test) | biLSTMClf | 0.289  $\pm$0.025 | 0.273  $\pm$0.018 | 0.280  $\pm$0.007 | 0.238  $\pm$0.006 | 0.798  $\pm$0.004 |
|  | GraphBind | **0.463**  $\boldsymbol{\pm}$**0.036** | **0.294**  $\boldsymbol{\pm}$**0.017** | **0.358**  $\boldsymbol{\pm}$**0.008** | **0.322**  $\boldsymbol{\pm}$**0.008** | **0.854**  $\boldsymbol{\pm}$**0.006** |

Table S3. Performance of GraphBind with different combinations of residue features on DNA-129_test. We report the average after having performed each experiment ten times.

| Features | Rec | Pre | F1 | MCC | AUC |
| --- | --- | --- | --- | --- | --- |
| PSSM | 0.631  $\pm$0.037 | 0.341  $\pm$0.027 | 0.441  $\pm$0.014 | 0.417  $\pm$0.010 | 0.900  $\pm$0.003 |
| HMM | 0.649  $\pm$0.034 | 0.398  $\pm$0.027 | 0.492  $\pm$0.013 | 0.468  $\pm$0.009 | 0.918  $\pm$0.005 |
| PSSM+HMM | 0.660  $\pm$0.028 | 0.406  $\pm$0.029 | 0.502  $\pm$0.015 | 0.478  $\pm$0.011 | 0.916  $\pm$0.006 |
| PSSM+HMM+SS | **0.686**  $\boldsymbol{\pm}$**0.032** | 0.413  $\pm$0.023 | 0.515  $\pm$0.013 | 0.494  $\pm$0.011 | **0.928**  $\boldsymbol{\pm}$**0.005** |
| PSSM+HMM+SS+AF | 0.676  $\pm$0.027 | **0.425**  $\boldsymbol{\pm}$**0.017** | **0.522**  $\boldsymbol{\pm}$**0.005** | **0.499**  $\boldsymbol{\pm}$**0.004** | 0.927  $\pm$0.006 |

Table S4. The performance of GraphBind trained on the nucleic acid-binding training sets without data augmentation. We report the average after having performed each experiment ten times.

| Dataset | Method | Rec | Pre | F1 | MCC | AUC |
| --- | --- | --- | --- | --- | --- | --- |
| DNA (DNA-129_Test) | No data augmentation | 0.588  $\pm$0.044 | **0.465**  $\boldsymbol{\pm}$**0.031** | 0.517  $\pm$0.012 | 0.487  $\pm$0.011 | 0.919  $\pm$0.005 |
|  | Data augmentation | **0.676**  $\boldsymbol{\pm}$**0.027** | 0.425  $\pm$0.017 | **0.522**  $\boldsymbol{\pm}$**0.005** | **0.499**  $\boldsymbol{\pm}$**0.004** | **0.927**  $\boldsymbol{\pm}$**0.006** |
| RNA (RNA-117_Test) | No data augmentation | 0.404  $\pm$0.051 | **0.306**  $\boldsymbol{\pm}$**0.020** | 0.346  $\pm$0.013 | 0.307  $\pm$0.014 | 0.849  $\pm$0.003 |
|  | Data augmentation | **0.463**  $\boldsymbol{\pm}$**0.036** | 0.294  $\pm$0.017 | **0.358**  $\boldsymbol{\pm}$**0.008** | **0.322**  $\boldsymbol{\pm}$**0.008** | **0.854**  $\boldsymbol{\pm}$**0.006** |

Table S5. The performance of GraphBind using the predicted protein structures from sequences. We report the average after having performed each experiment ten times.

| Dataset | TM-score^a^ | Rec | Pre | F1 | MCC | AUC |
| --- | --- | --- | --- | --- | --- | --- |
| DNA (DNA-129_Test) | 0.544 | 0.439  $\pm$0.035 | 0.310  $\pm$0.021 | 0.362  $\pm$0.011 | 0.320  $\pm$0.011 | 0.816  $\pm$0.010 |
| RNA (RNA-117_Test) | 0.458 | 0.303  $\pm$0.039 | 0.171  $\pm$0.008 | 0.218  $\pm$0.009 | 0.168  $\pm$0.010 | 0.718  $\pm$0.007 |

^a^ The TM-score of a test set is the average TM-score between the predicted structure and the experimental structure.

Table S6. Summary of the five benchmark ligand datasets.

| Type | Dataset | N_protein_^a^ | N_pos_^b^ | N_neg_^c^ | PNratio^d^ |
| --- | --- | --- | --- | --- | --- |
| Ca^2+^ | CA-1022_Train | 1,022 | 4,830 | 255,917 | 0.019 |
|  | CA-515_Test | 515 | 2,958 | 186,678 | 0.016 |
| Mn^2+^ | MN-440_Train | 440 | 1,931 | 150,229 | 0.013 |
|  | MN-144_Test | 144 | 612 | 50,838 | 0.012 |
| Mg^2+^ | MG-1194_Train | 1,194 | 4,147 | 320,736 | 0.013 |
|  | MG-651_Test | 651 | 2,321 | 244,088 | 0.010 |
| ATP | ATP-388_Train | 388 | 5,657 | 142,086 | 0.040 |
|  | ATP-41_Test | 41 | 674 | 14,149 | 0.048 |
| HEME | HEM-175_Train | 175 | 3,851 | 44,477 | 0.087 |
|  | HEM-96_Test | 96 | 2,012 | 26,341 | 0.076 |

^a^ Number of proteins.

^b^ Number of binding residues.

^c^ Number of nonbinding residues.

^d^ PNratio = N_pos_/N_neg_.

Table S7. Performance comparison of GraphBind with other state-of-the-art methods on test sets of metal ions (i.e. Ca^2+^, Mn^2+^ and Mg^2+^) and biologically relevant molecules (i.e. ATP and HEME). N/A means the metric cannot be measured since the webserver or software only outputs binary scores. We report the average after having performed each experiment ten times.

| Dataset | Method | Rec | Pre | F1 | MCC | P-values of MCC | AUC | P-values of AUC |
| --- | --- | --- | --- | --- | --- | --- | --- | --- |
| Ca^2+^  (CA-515_Test) | TargetS | 0.174 | 0.506 | 0.259 | 0.291 | 7.78$\times$10^-11^ | N/A | N/A |
|  | S-SITE^a^ | 0.303 | 0.124 | 0.176 | 0.174 | 3.66$\times$10^-15^ | 0.661 | 4.78$\times$10^-14^ |
|  | COACH^a^ | 0.297 | 0.162 | 0.210 | 0.203 | 3.94$\times$10^-13^ | 0.671 | 6.85$\times$10^-14^ |
|  | IonCom^b^ | 0.190 | 0.331 | 0.241 | 0.242 | 1.09$\times$10^-10^ | 0.717 | 2.39$\times$10^-12^ |
|  | DELIA^c^ | 0.182 | 0.556 | 0.274 | 0.313 | 1.84$\times$10^-10^ | 0.795 | 3.42$\times$10^-11^ |
|  | **GraphBind** | **0.325**  $\boldsymbol{\pm}$**0.031** | **0.563**  $\boldsymbol{\pm}$**0.040** | **0.410**  $\boldsymbol{\pm}$**0.017** | **0.420**  $\boldsymbol{\pm}$**0.011** | N/A | **0.863**  $\boldsymbol{\pm}$**0.012** | N/A |
| Mn^2+^  (MN-144_Test) | TargetS | 0.395 | 0.499 | 0.441 | 0.438 | 1.91$\times$10^-10^ | N/A | N/A |
|  | S-SITE | 0.369 | 0.526 | 0.434 | 0.435 | 4.11$\times$10^-10^ | 0.817 | 1.32$\times$10^-10^ |
|  | COACH | 0.562 | 0.272 | 0.367 | 0.381 | 4.44$\times$10^-11^ | 0.821 | 3.47$\times$10^-11^ |
|  | IonCom | 0.531 | 0.495 | 0.512 | 0.506 | 8.03$\times$10^-5^ | 0.872 | 3.31$\times$10^-9^ |
|  | DELIA | 0.513 | **0.632** | 0.566 | 0.565 | 2.31$\times$10^-4^ | 0.903 | 7.66$\times$10^-8^ |
|  | **GraphBind** | **0.563**  $\boldsymbol{\pm}$**0.044** | 0.626  $\pm$0.030 | **0.591**  $\boldsymbol{\pm}$**0.012** | **0.588**  $\boldsymbol{\pm}$**0.011** | N/A | **0.951**  $\boldsymbol{\pm}$**0.006** | N/A |
| Mg^2+^  (MG-651_Test) | TargetS | 0.154 | 0.449 | 0.229 | 0.259 | 4.76$\times$10^-9^ | N/A | N/A |
|  | S-SITE | 0.243 | 0.132 | 0.171 | 0.169 | 2.03$\times$10^-14^ | 0.682 | 1.57$\times$10^-15^ |
|  | COACH | **0.273** | 0.124 | 0.171 | 0.169 | 1.34$\times$10^-12^ | 0.675 | 1.19$\times$10^-15^ |
|  | IonCom | 0.155 | 0.317 | 0.208 | 0.217 | 7.49$\times$10^-11^ | 0.685 | 1.36$\times$10^-15^ |
|  | DELIA | 0.143 | **0.562** | 0.228 | 0.280 | 3.80$\times$10^-8^ | 0.780 | 1.69$\times$10^-11^ |
|  | **GraphBind** | 0.259  $\pm$0.013 | 0.410  $\pm$0.026 | **0.317**  $\boldsymbol{\pm}$**0.006** | **0.320**  $\boldsymbol{\pm}$**0.007** | N/A | **0.827**  $\boldsymbol{\pm}$**0.007** | N/A |
| ATP^j^  (ATP-41_Test) | TargetS | 0.516 | 0.689 | 0.590 | 0.580 | 4.74$\times$10^-7^ | N/A | N/A |
|  | S-SITE | 0.570 | 0.505 | 0.536 | 0.513 | 2.92$\times$10^-10^ | 0.801 | 2.10$\times$10^-9^ |
|  | COACH | 0.632 | 0.703 | 0.666 | 0.652 | 1.84$\times$10^-3^ | N/A | 3.51$\times$10^-11^ |
|  | ATPbind^d^ | 0.631 | 0.756 | 0.688 | 0.677 | 0.7009 | 0.915 | 4.31$\times$10^-9^ |
|  | DELIA | **0.642** | **0.758** | **0.695** | **0.685** | N/A | **0.947** | N/A |
|  | **GraphBind** | 0.603  $\pm$0.037 | 0.666  $\pm$0.035 | 0.631  $\pm$0.012 | 0.616  $\pm$0.011 | 1.05$\times$10^-6^ | 0.939  $\pm$0.006 | 3.78$\times$10^-3^ |
| HEME  (HEM-96_Test) | TargetS | 0.493 | **0.756** | 0.597 | 0.588 | 3.23$\times$10^-8^ | N/A | N/A |
|  | S-SITE | 0.619 | 0.580 | 0.599 | 0.568 | 1.44$\times$10^-9^ | 0.813 | 2.72$\times$10^-13^ |
|  | COACH | 0.677 | 0.403 | 0.505 | 0.476 | 9.78$\times$10^-13^ | 0.835 | 6.19$\times$10^-11^ |
|  | DELIA | 0.648 | 0.660 | 0.654 | 0.628 | 5.12$\times$10^-6^ | 0.951 | 4.41$\times$10^-8^ |
|  | **GraphBind** | **0.775**  $\boldsymbol{\pm}$**0.032** | 0.610  $\pm$0.026 | **0.682**  $\boldsymbol{\pm}$**0.008** | **0.661**  $\boldsymbol{\pm}$**0.008** | N/A | **0.962**  $\boldsymbol{\pm}$**0.003** | N/A |

^a^ Results are computed using the standalone program at <https://zhanglab.ccmb.med.umich.edu/COACH/>.

^b^ Results are computed using the standalone program at <https://zhanglab.ccmb.med.umich.edu/IonCom/>.

^c^ Results of DELIA are directly from the original paper(1).

^d^ Results for ATP-binding residues are directly from the original paper(2).

**Supplementary Figures**


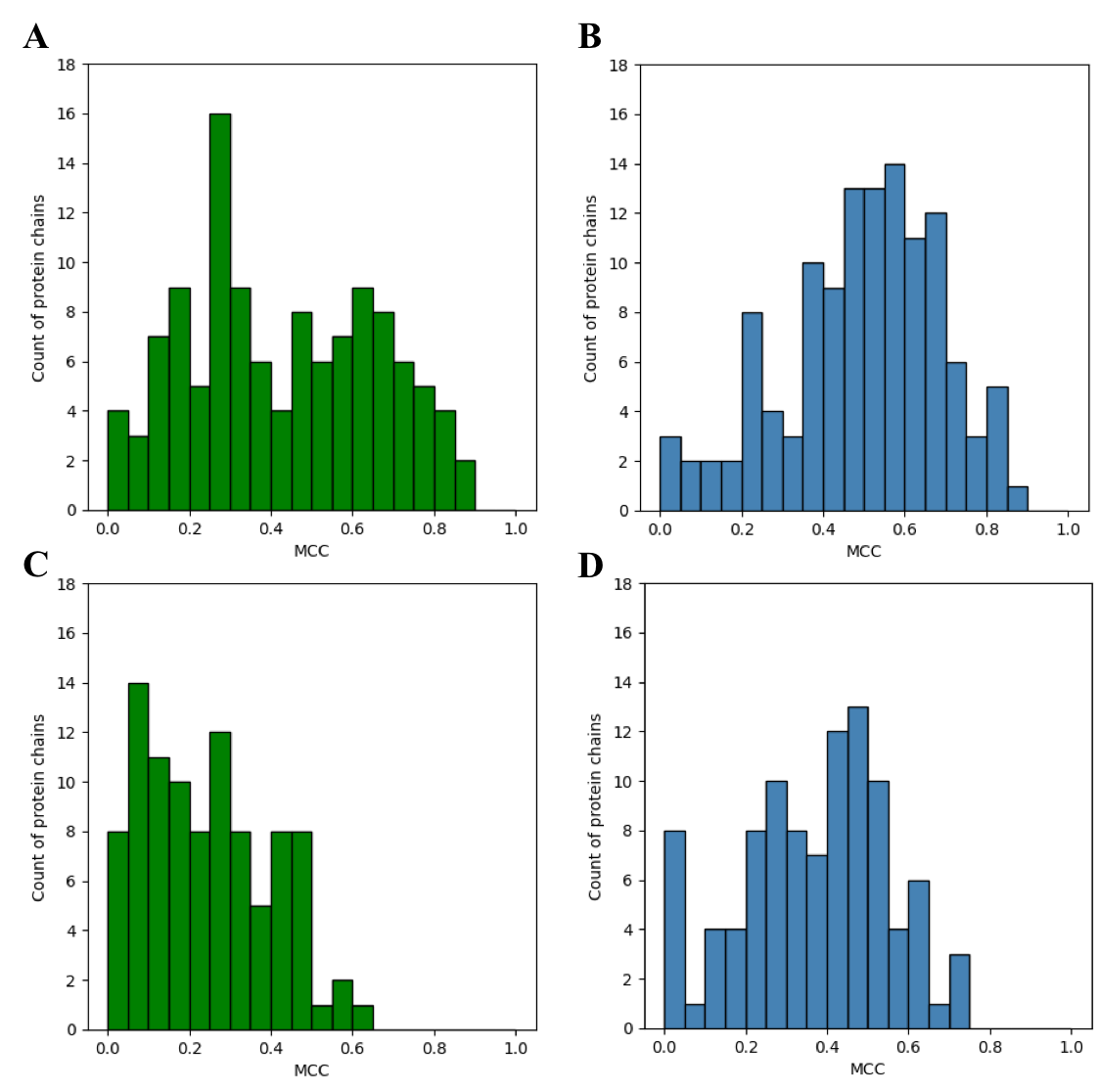


Figure S1. The distribution of predicted MCCs of each protein chain in the test set. Figures A, B, C and D corresponds to DNABind on DNA-129_Test, GraphBind on DNA-129_Test, NucleicNet on RNA-117_Test and GraphBind on RNA-117_Test, respectively.

**REFERENCES**

1. Xia, C.-Q., Pan, X. and Shen, H.-B. (2020) Protein–ligand binding residue prediction enhancement through hybrid deep heterogeneous learning of sequence and structure data. *Bioinformatics*, **36**, 3018-3027.

2. Hu, J., Li, Y., Zhang, Y. and Yu, D.-J. (2018) ATPbind: accurate protein–ATP binding site prediction by combining sequence-profiling and structure-based comparisons. *Journal of chemical information and modeling*, **58**, 501-510.
